# Effect of Calcination Temperature on the Reactivity of Lithium Slag Powder as a Supplementary Cementitious Material

**DOI:** 10.3390/ma19163546

**Published:** 2026-08-21

**Authors:** Yoo Jung Hwang, Young-Cheol Choi

**Affiliations:** Department of Civil and Environmental Engineering, Gachon University, Seongnam-si 13120, Gyeonggi-do, Republic of Korea; yoojung200311@gachon.ac.kr

**Keywords:** lithium slag powder, thermal activation, pozzolanic reactivity, R3 pozzolanic test, Chapelle test, alkaline leaching

## Abstract

Lithium slag powder (LSP), a by-product of lithium extraction, has attracted increasing interest as a supplementary cementitious material (SCM) due to its aluminosilicate-rich composition and growing availability. However, its limited intrinsic reactivity constrains direct use in cementitious systems. This study systematically investigates the effect of calcination temperature on the physicochemical properties, pozzolanic reactivity, and cement hydration performance of LSP. LSP was thermally treated at 300–900 °C, and structural and morphological changes were characterized using X-ray diffraction, scanning electron microscopy, and Fourier-transform infrared spectroscopy. The reactivity of calcined LSP was quantitatively assessed through isothermal calorimetry (R3 test), thermogravimetric and derivative thermogravimetric analysis. Chapelle testing, leaching tests, and compressive strength measurements of cement mortars. Controlled calcination was found to enhance the intrinsic reactivity and pozzolanic activity of LSP, resulting in improved long-term mechanical performance. The findings provide mechanistic insights and practical guidance for the sustainable use of lithium slag as an SCM in cement-based materials.

## 1. Introduction

The cement industry is widely recognized as one of the most carbon-intensive industrial sectors due to its reliance on large quantities of natural resources—such as limestone and silica—and energy-intensive, high-temperature clinkerization processes. Previous studies report that the production of one ton of cement clinker emits approximately one ton of CO_2_, contributing to the nearly 1.6 billion tons of CO_2_ released annually by the global cement industry [1,2]. Overall, cement production accounts for approximately 5–8% of global greenhouse gas emissions, primarily from limestone calcination and fossil fuel combustion. In addition, substantial amounts of atmospheric pollutants—including nitrogen oxides, sulfur dioxide, and particulate matter—are generated, imposing a significant environmental burden [3]. In response, extensive efforts have been made worldwide to reduce carbon emissions and promote resource circularity in the cement industry. In particular, the use of industrial by-products and waste as supplementary cementitious materials (SCMs) has gained increasing attention [4]. Conventional SCMs—such as fly ash (FA) from coal-fired power plants and ground granulated blast furnace slag (GGBS) from the steel industry—have been incorporated at various replacement levels in cement and concrete. However, at high replacement ratios, these traditional SCMs face persistent limitations, including regional supply imbalances, quality variability, limited availability, and reduced reactivity. Consequently, from a long-term sustainability perspective, the development and large-scale implementation of alternative SCMs has become increasingly urgent [5,6,7].

Recently, considerable research has focused on using silicate- and aluminosilicate-based solid wastes as SCMs to reduce the energy consumption and CO_2_ emissions associated with cement production [8]. These approaches are considered effective for conserving natural resources and mitigating environmental impacts [8]. Lithium slag powder (LSP), a solid by-product generated during lithium refining, is rich in silica and alumina and contains significant amounts of calcium, iron, and other metal oxides, indicating strong potential as a cementitious substitute [9]. Numerous studies have reported that LSP can enhance the mechanical performance of concrete, highlighting its promise as an SCM candidate [2,5]. With the rapid growth of electric vehicle and energy storage markets, global lithium demand has surged, leading to increased LSP generation. Thus, valorizing LSP is an important strategy for both waste reduction and the development of sustainable construction materials [5,8,10]. During production, LSP typically undergoes high-temperature treatment followed by rapid cooling, partially forming amorphous aluminosilicate phases [5,11]. Similar to FA ash, these mineralogical characteristics impart pozzolanic reactivity to LSP, enabling it to modify long-term hydration products and contribute to strength development when incorporated into cementitious systems [6].

Previous studies have reported conflicting observations regarding the role of LSP in cement hydration. Some researchers have indicated that LSP promotes the formation of specific hydration products, whereas others have observed retardation of early-age hydration [12,13]. When properly ground, LSP can act as a microfiller in concrete, interacting with hydration products to refine the microstructure and enhance material properties [14,15,16]. The large-scale stockpiling of slag generated during lithium compound production poses significant environmental concerns [5]. Currently, LSP is predominantly disposed of in landfills or stored long-term, further exacerbating the environmental burden [17]. Consequently, there is a clear need to establish technical strategies that enable effective waste management and meaningful utilization of LSP in the cement industry. According to Wang et al. [18], thermal treatment effectively enhances LSP reactivity by altering its crystal structure, inducing partial amorphization, and modifying the oxidation states of certain constituents. Their results demonstrated that thermal activation increases LSP solubility in cementitious pastes, yielding LSP–cement mortars with higher hydration product content and improved mechanical performance. Thermal activation is widely employed to improve the reactivity of low-activity solid wastes [19]. When exposed to elevated temperatures, aluminosilicate minerals undergo dehydroxylation and structural rearrangement, resulting in reduced crystallinity and increased amorphous content [20,21]. These thermochemical transformations significantly enhance material reactivity and broaden the potential applicability of LSP in binder systems [22]. Accordingly, recent studies have increasingly investigated the thermal activation of LSP for use in cement and concrete materials.

Liu et al. and Wang et al. [10,23] reported that the combined application of thermal and chemical activation significantly enhanced the reactivity of lithium slag in geopolymer production, increasing the proportion of active amorphous phases from 17.3% to 50.7%. Similarly, Luo et al. [24] demonstrated that calcination pretreatment improved the compressive strength of lithium slag-based geopolymers, showing a strong correlation between reactivity and strength development. However, most studies have focused on geopolymer synthesis under highly alkaline conditions, while fundamental aspects—such as mix design, reaction product composition, and microstructural evolution of thermally treated LSP used as the sole precursor—remain insufficiently understood [25,26,27]. Moreover, systematic investigations into how temperature-dependent structural transformations of LSP influence cement hydration mechanisms are limited. Although some studies have examined the inclusion of thermally treated LSP in cementitious systems, the formation pathways of hydration products and early-age reaction kinetics have not been clearly elucidated. Therefore, in-depth research is required to clarify the effect of LSP’s thermally induced structural changes on cement hydration. Such understanding is essential for establishing a scientific basis for using LSP as an SCM and carries significant implications for resource recycling and the broader adoption of alternative binders [19].

Although previous studies have demonstrated that thermal treatment can enhance the reactivity of lithium slag, most investigations have focused on geopolymer or highly alkaline activation systems, and the temperature-dependent relationship between structural transformation, intrinsic pozzolanic reactivity, Portland cement hydration, and mechanical performance remains insufficiently established. Therefore, the novelty of this study lies in the systematic comparison of six LSP conditions—uncalcined LSP and LSP calcined at 300, 500, 700, 800, and 900 °C—using an integrated multiscale evaluation framework. Mineralogical, chemical, and morphological transformations were correlated with intrinsic reactivity determined through the R3 test, bound-water and Ca(OH)_2_ analyses, the Chapelle test, and alkaline leaching, as well as with hydration kinetics and compressive-strength development in Portland cement systems. This comprehensive approach enables the effects of thermal activation to be distinguished from dilution, sulfate-related reactions, and high-temperature recrystallization, thereby identifying the calcination range that most effectively improves the performance of LSP as a supplementary cementitious material.

## 2. Materials and Methods

### 2.1. Materials

In this study, the influence of LSP on cement hydration behavior and reactivity was evaluated and compared with that of a conventional SCM. Ordinary Portland cement (OPC)—conforming to ASTM C150/C150M–20 [28] Type I and manufactured by Asia Cement Co., Ltd. (Seoul, Republic of Korea)—was used as the primary binder. FA—produced by the Yeongheung Power Plant (Incheon, Republic of Korea)—was selected as the reference SCM. LSP is characterized by its pozzolanic reactivity, agglomerated fine-particle structure, and favorable filler effect, making it a promising cementitious additive [17,29]. The LSP used in this study was a by-product of lithium production obtained from Pohang Iron and Steel Co., Ltd. (Pohang, Republic of Korea) and mainly consisted of silicon dioxide (SiO_2_), aluminum oxide (Al_2_O_3_), and calcium oxide (CaO). Table 1 lists the chemical compositions and densities of the raw materials. The chemical compositions were determined by using X-ray fluorescence (XRF) spectrometer (Rigaku ZSX Primus II, Rigaku Corporation, Tokyo, Japan). As shown in Table 1, FA and LSP have similar major chemical constituents. However, LSP has a lower Fe_2_O_3_ content and higher SO_3_ content than FA. Such compositional differences may influence cement hydration reactions and strength development. In addition, compared with OPC, LSP contains significantly higher amounts of SiO_2_ and Al_2_O_3_, suggesting a strong potential for utilization as a SCM through pozzolanic reactions [11]. Nevertheless, the CaO content of LSP is relatively low compared to that of other cementitious materials, limiting its use as a standalone cement replacement [1].

**Figure 1:**
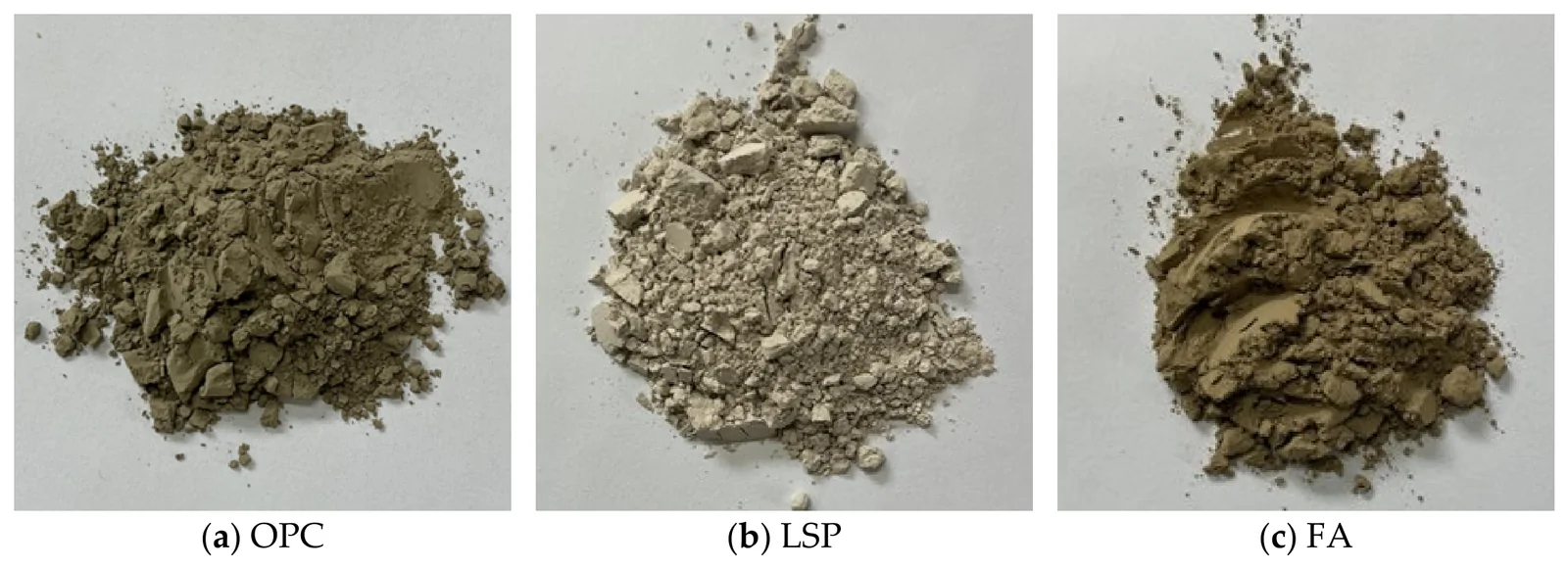
Optical images of the raw materials.

**Figure 2:**
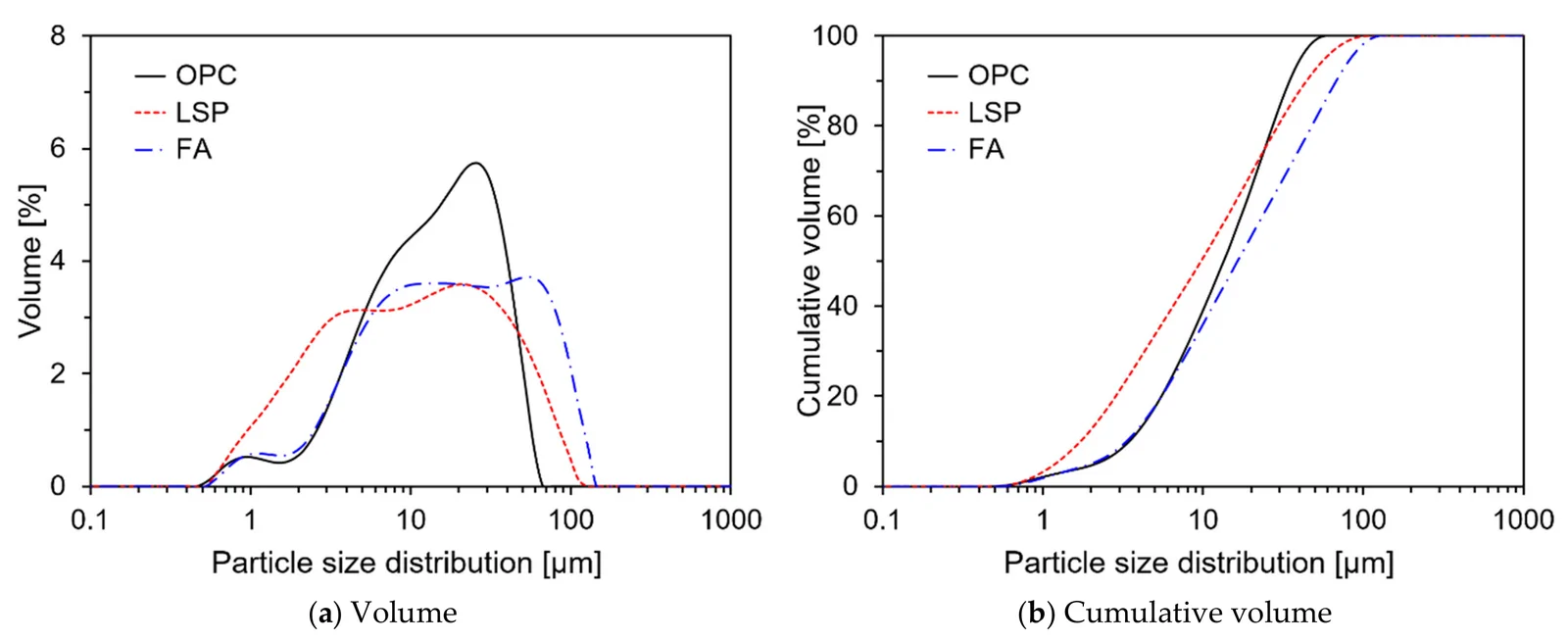
Particles distributions of OPC, LSP, GGBS, and FA.

Figure 1 shows optical images of the raw materials, and Figure 2 shows their particle size distributions. The particle size distributions of the raw materials were measured in accordance with ASTM C115-10 [30] using a laser diffraction particle size analyzer (LS 230, Beckman Coulter, CA, USA). The mean particle size of OPC was 17.7 µm, whereas those of FA and LSP were 28.7 and 18.3 µm, respectively. The particle size distribution of OPC exhibited a monomodal pattern, whereas those of FA and LSP displayed bimodal distributions. Cement hydration behavior is influenced by the physical characteristics of blended materials, including particle size and morphology, as well as their chemical properties. Consequently, variations in particle size distribution and material characteristics between OPC, FA, and LSP were predicted to affect hydration kinetics and the overall hydration process.

**Figure 3:**
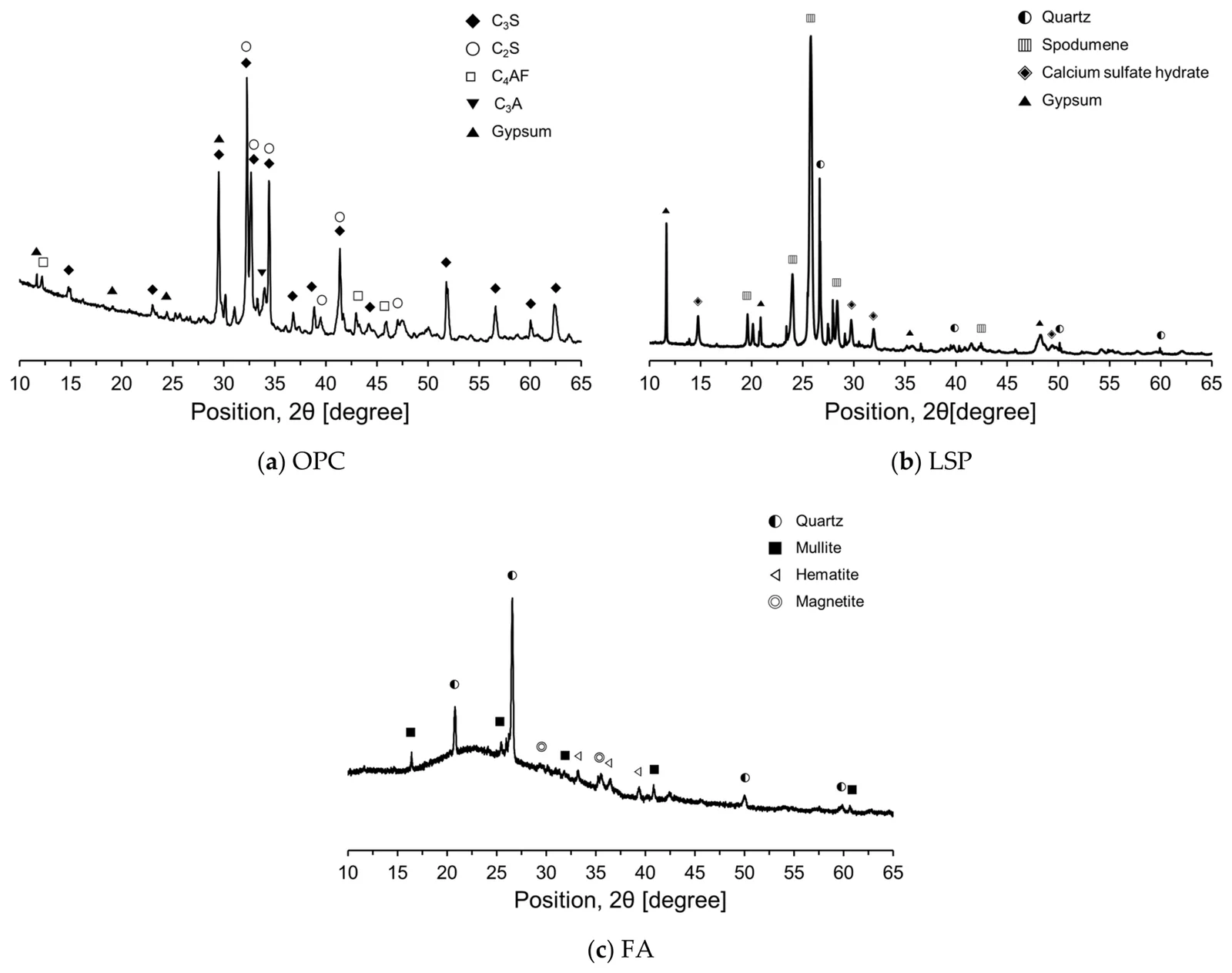
XRD patterns of the raw materials.

Figure 3 shows the X-ray diffraction (XRD) patterns of the raw materials. X-ray diffraction (XRD) patterns were obtained using a PANalytical X’Pert Pro MPD diffractometer (Malvern PANalytical, Almelo, The Netherlands). As expected, the primary mineral phases of OPC are C_3_A, C_2_S, C_3_S, and C_4_AF (Figure 3a). The XRD analysis of LSP indicates that LSP is mainly composed of spodumene (LiAl(SiO_3_)_2_), quartz (SiO_2_), calcium sulfate hydrates, and gypsum (Figure 3b). Lithium is generally extracted from minerals such as spodumene and lepidolite, which undergo roasting at temperatures of approximately 850–1150 °C to form highly reactive and amorphous β-spodumene [11]. The phase transformation of spodumene is associated with the substitution of Si atoms with Al atoms, which results in the formation of a tetrahedral framework. During this process, charge balance is maintained through the incorporation of lithium ions that compensate for the negative charge generated by Al substitution [17]. The lithium slag remaining after lithium extraction contains a substantial proportion of amorphous aluminosilicate phases, which have been reported to exhibit effective pozzolanic reactivity when used as a cementitious admixture [10]. The carbonate minerals observed in LSP are presumed to originate from the lithium carbonate production process [11]. The principal crystalline phases of FA include quartz (SiO_2_), mullite (3Al_2_O_3_·2SiO_2_), hematite (Fe_2_O_3_), and magnetite (Fe_3_O_4_) [31,32]. The presence of a broad amorphous hump in the range of 20–35° (2θ) (Figure 3c) confirms the existence of a significant amorphous phase in FA [33]. In general, the relative proportions of crystalline and amorphous phases in FA are strongly influenced by the type of fuel used and the operating conditions and combustion characteristics of the thermal power plant [34].

**Figure 4:**
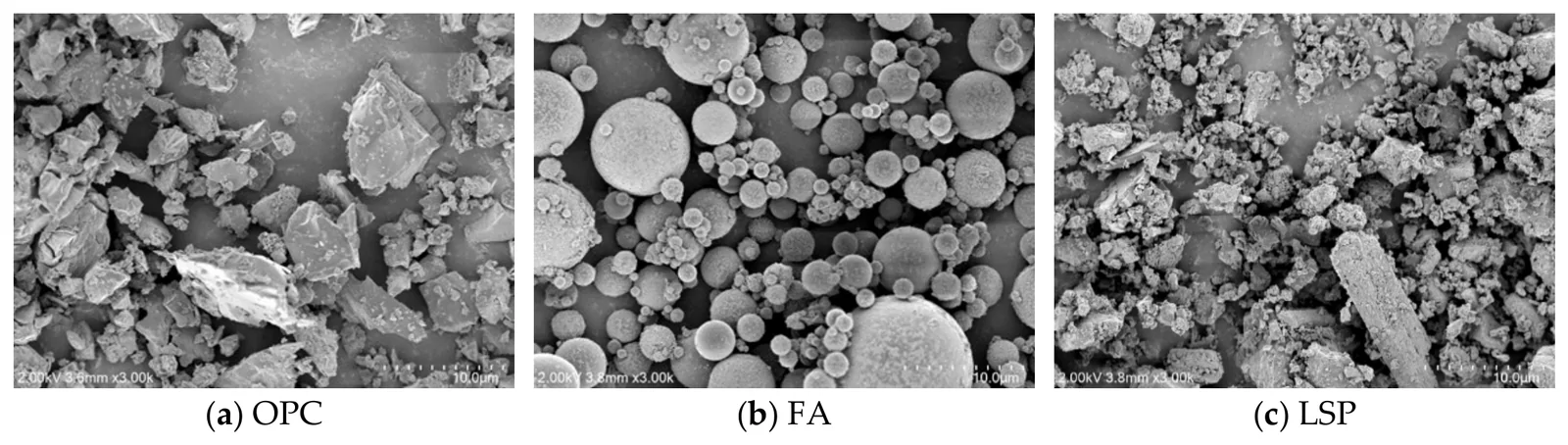
SEM images of the raw materials.

Figure 4 shows scanning electron microscopy (SEM) images of the raw materials used in this study. The SEM micrographs reveal that OPC has a gray appearance and an irregular surface texture. As shown in Figure 4b, FA consists predominantly of irregular spherical particles at the micrometer scale. These hollow, glassy spheres are typically formed during coal combustion, in which molten aluminosilicates encapsulate gas bubbles or unburned carbon, resulting in lightweight and relatively inert structures [35,36]. In contrast, LSP appears as irregular block-shaped particles [33,37]. The distinctive morphology of LSP can be attributed to its heterogeneous mineralogical composition, which mainly comprises layered bulk spodumene, spherical spodumene, and rod-like gypsum phases [17]. LSP particles exhibit a wide range of shapes, with finer particles agglomerating to form larger, irregular clusters, within which pores are commonly observed. In addition, some particles exhibit rod-like CaSO_4_·2H_2_O morphologies or present as granular SiO_2_ phases [35,38].

### 2.2. Test Methods

To determine the mineralogical compositions of the raw materials, XRD analysis was conducted using an X-ray diffractometer (X’Pert Pro MPD, Empyrean, Malvern Panalytical, Almelo, The Netherlands) equipped with an X’Celerator detector. Measurements were performed using CuKα radiation (λ = 1.54 Å) and operated at 40 kV and 40 mA. Continuous scanning was conducted from 10 to 65° (2θ) with a step size of 0.01° and counting time of 1 s per step. SEM was employed to examine the morphology of the raw materials using an SU8600 instrument (Hitachi, Tokyo, Japan). For SEM observation, the samples were mounted on conductive adhesive, followed by vacuum treatment and platinum coating prior to imaging. Thermogravimetric analysis (TGA) was performed using an STA 8122 thermal analyzer (Rigaku Corporation, Tokyo, Japan) [39]. Prior to analysis, specimen hydration was stopped by vacuum treatment, followed by immersion in isopropanol. The samples were then dried at 40 °C for 24 h and pulverized using a planetary mill (Pulverisette 5/2, Fritsch, Idar-Oberstein, Germany). TGA measurements were conducted up to 1000 °C at a heating rate of 10 °C/min to record mass changes. Alumina pans with a capacity of 30 μL were used, and a nitrogen (N_2_) atmosphere was maintained by flushing at a flow rate of 100 mL/min. To investigate the effect of LSP on the hydration behavior of OPC, isothermal calorimetry tests were conducted using a TAM Air isothermal calorimeter (TA Instruments, Sollentuna, Sweden). The TAM Air system operates in the milliwatt range and comprises eight independent measurement channels [35]. Approximately 10 g of paste—prepared according to the designed mix proportions—was sealed in glass ampoules and monitored at 20 °C for 72 h.

The pozzolanic reactivities of LSP and FA were evaluated using the R3 test in accordance with ASTM C1897 [40].Three complementary approaches were employed: heat release, bound water content, and Ca(OH)_2_ consumption. The heat-based R3 test was performed according to ASTM C1897 Method A using a TAM Air isothermal calorimeter (TA Instruments, DE, USA). The paste mixture comprised 20 g of pozzolanic material, 60 g of Ca(OH)_2_, 10 g of CaCO_3_, and 108 g of alkaline solution containing 0.43 g/L KOH and 2.16 g/L K_2_SO_4_ dissolved in deionized water. The heat evolution was continuously recorded at 40 °C for 168 h. The results were normalized and expressed as the cumulative heat release per gram of pozzolanic material. Pozzolanic reactivity was further assessed by measuring the bound water content and Ca(OH)_2_ consumption using paste specimens prepared identically to those used in the heat-release R3 test. After curing at 40 °C for 7 d, the specimens were dried for 24 h. The surface layer exposed to air was discarded, and the remaining material was ground using an agate mortar. The powdered samples were dried at 40 °C and subsequently heated in a furnace at 350 °C for 2 h, followed by cooling to determine mass loss. To minimize errors associated with the dehydroxylation of portlandite above 400 °C, the maximum temperature was limited to 350 °C [41]. This procedure was based on the methodology proposed by Avet et al. [42]. The Ca(OH)_2_ content was quantified using TG–DTG analysis. Typically, Ca(OH)_2_ decomposes between 310 and 480 °C, whereas portions of synthetic C–S–H gel free of Ca(OH)_2_ and CaCO_3_ decompose in the range of 40–300 °C. In mixed systems containing both synthetic C–S–H gel and Ca(OH)_2_, a distinct Ca(OH)_2_ decomposition peak is observed, along with a CaCO_3_ decomposition peak at higher temperatures [43].

**Figure 5:**
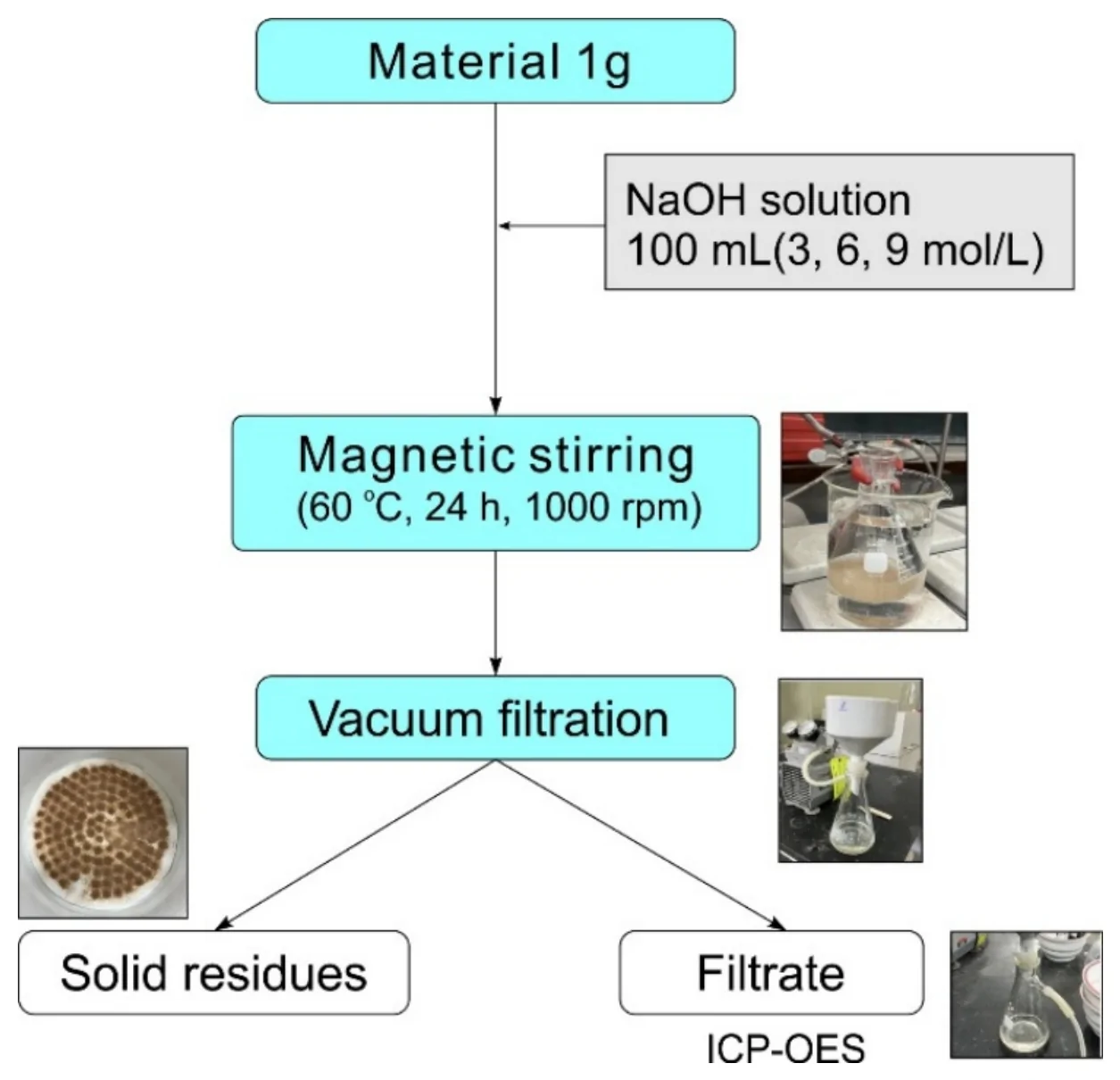
LSP leaching process.

To analyze the selective dissolution behavior of lithium slag under alkaline conditions, leaching tests were conducted using sodium hydroxide (NaOH) solution, as shown in Figure 5 [44]. One gram of dried LSP or FA was placed in a 250 mL flask and mixed with 100 mL of NaOH aqueous solution at concentrations of 3, 6, and 9 mol/L. NaOH solution was prepared using analytical grade reagents and deionized water. The suspension was stirred at 1000 rpm using a magnetic stirrer and maintained at 60 °C for 24 h in a temperature-controlled water bath. After leaching, the solid and liquid phases were separated by vacuum filtration. The filtrates obtained after leaching were analyzed using inductively coupled plasma–optical emission spectroscopy (Optima 8300, PerkinElmer, Waltham, MA, USA) to determine the concentrations of Ca, Al, and Si as functions of NaOH solution concentration. The dissolution ratio of each element was calculated by comparing the elemental concentrations in the filtrates with the corresponding elemental contents of the original raw materials.

**Figure 6:**
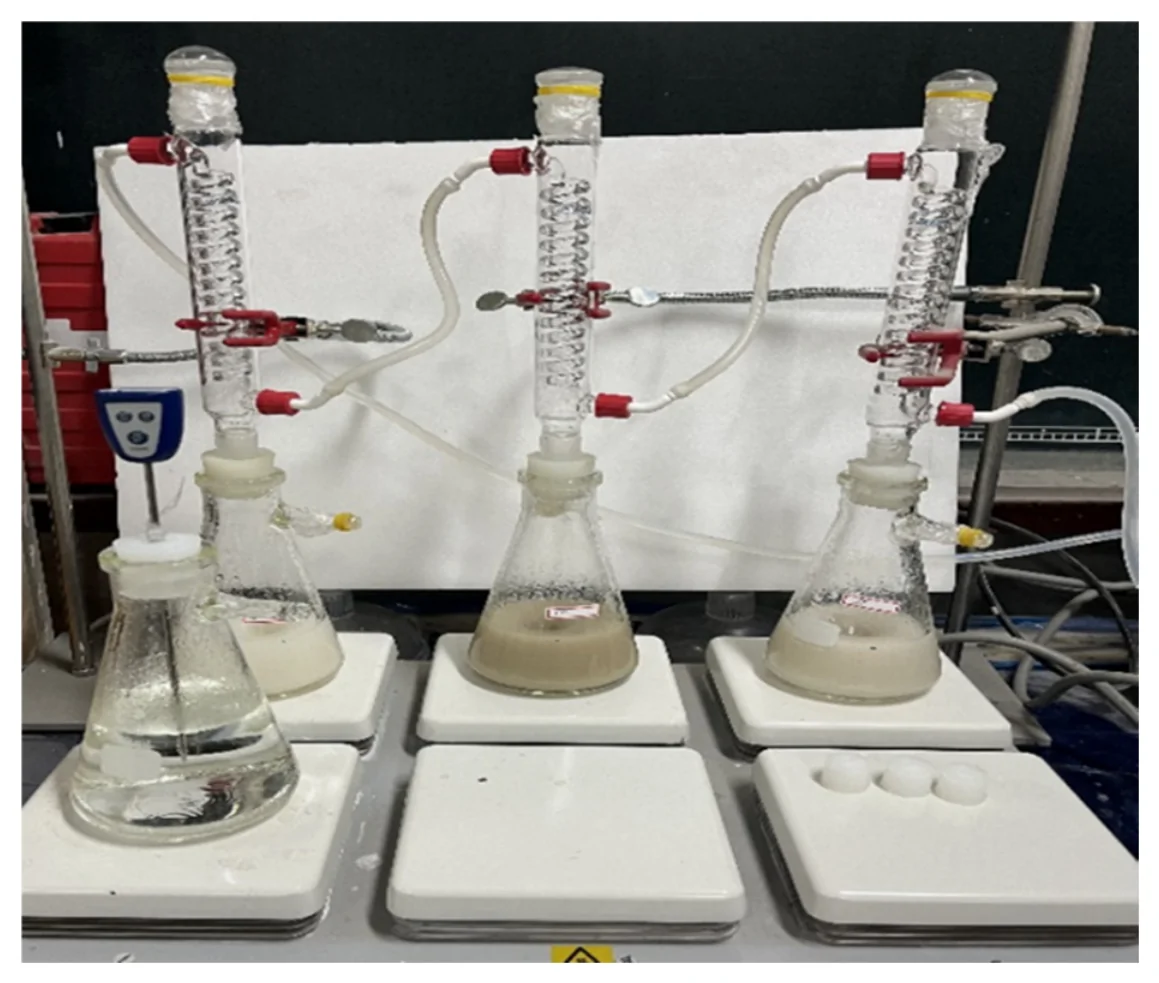
Photograph of the Chapelle test set-up.

The pozzolanic reactivities of the SCMs were quantitatively evaluated using the Chapelle test method in accordance with NF P18-513 [45] (Figure 6). This test quantifies the amount of calcium hydroxide consumed by a given material under controlled conditions. For this test, 1 g of the supplementary material and 2 g of CaO were placed in a conical flask containing 250 mL of deionized water. The flask was equipped with a reflux condenser and maintained at 90 °C for 16 h under continuous stirring. After completion of the reaction, the suspension was cooled to room temperature, to which a 250 mL solution—prepared by dissolving 60 g of saccharose in 250 mL of deionized water—was added, followed by additional stirring for 30 min. Subsequently, 200 mL of the mixture was filtered, and 25 mL of the filtrate was titrated with 0.1 N HCl. The amount of Ca(OH)_2_ consumed was calculated as the difference in HCl consumption between the test sample and a reference sample without supplementary material. The pozzolanic reactivity was then calculated based on the amount of calcium hydroxide consumed per unit mass of SCM.

The compressive strength of the mortar specimens was tested in accordance with ISO 679 [46]. The mortar mixtures were prepared with a cement-to-water-to-sand mass ratio of 2:1:6. LSP and FA were used to replace 30 vol.% of the cement. The compressive strength of all mixtures was measured at 3, 7, 28, and 91 days. The two halves of the prism specimens fractured during the flexural strength test, providing a total of six specimens, were used for the compressive strength test. The compressive strength for each mixture and curing age was determined as the average value of the six specimens. To evaluate the hydration reactivity of LSP at different calcination temperatures, paste specimens were prepared using the same mixture proportions as those of the mortar specimens, but without sand, and were subjected to the relevant tests. The R3 test, bound-water and Ca(OH)_2_ analyses, Chapelle test, alkaline leaching test, and hydration kinetics measurements were each performed in triplicate under identical conditions, and the mean values of the three measurements were reported.

## 3. Results and Discussion

### 3.1. Calcination of LSP

**Figure 7:**
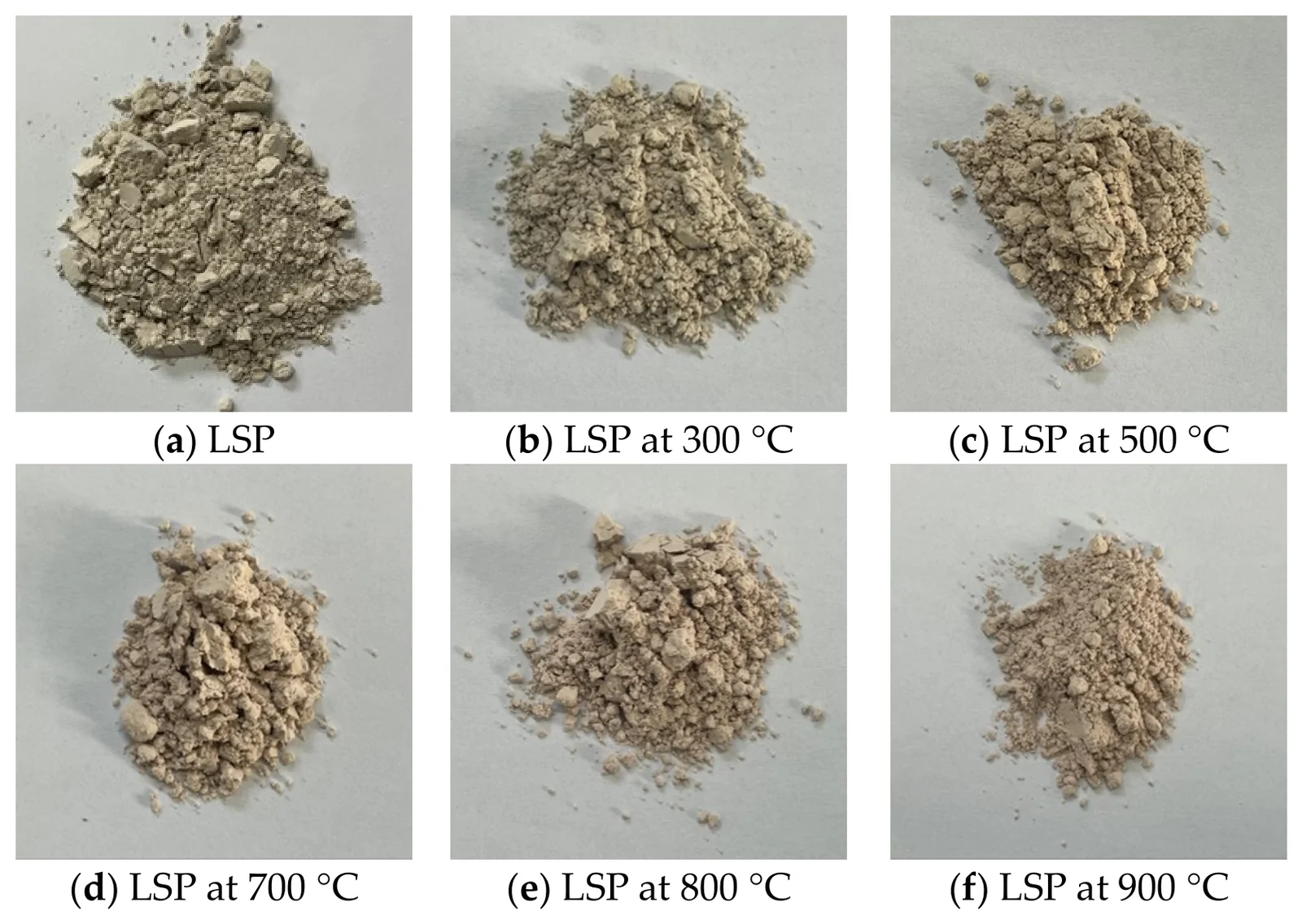
Optical images of LSP as a function of calcination.

In this study, the reactivity of LSP as a function of calcination temperature was investigated using an electric furnace manufactured by Daihan Scientific (Wonju, Republic of Korea). Room-temperature LSP was placed in the furnace and heated to the target calcination temperatures (300, 500, 700, 800, and 900 °C) at a controlled heating rate of 1 °C/min. Each target temperature was maintained for 4 h, after which the samples were allowed to cool naturally for approximately 1.5 h to room temperature. Figure 7 shows optical images of LSP calcined at different temperatures. LSP exhibits a pronounced color change with increasing calcination temperature, which is closely associated with changes in the oxidation state of iron-containing phases. As the thermal activation temperature increases, the originally light-colored LSP gradually develops a reddish hue. This color evolution is attributed to the oxidation of iron ions at elevated temperatures that leads to the formation of Fe_2_O_3_, which imparts the characteristic red coloration [18].

Table 2 summarizes the chemical composition of LSP calcined at different temperatures, as determined via XRF. The principal constituents of LSP are Al_2_O_3_, SiO_2_, SO_3_, CaO, Fe_2_O_3_, K_2_O, MgO, P_2_O_5_, MnO, Rb_2_O, TiO_2_, ZnO, and Cr_2_O_3_. With increasing calcination temperature, the SiO_2_ content gradually increases from 54.42 to 57.10 wt%, and Al_2_O_3_ exhibits a similar increasing trend. These compositional changes reflect dehydroxylation and structural reorganization within the aluminosilicate framework of lithium slag and are consistent with the known thermal behavior of spodumene and other related minerals [18,47]. Al_2_O_3_ plays a complex role in the decomposition behavior of CaSO_4_ during thermal treatment. In the initial stage, Al_2_O_3_ reacts with CaO, promoting the decomposition of CaSO_4_. However, as calcination proceeds, CaAl_2_O_4_ formation leads to secondary reactions with CaSO_4_ and CaO, resulting in the formation of the high-temperature stable phase Ca_4_Al_6_SO_16_. Ca_4_Al_6_SO_16_ formation tends to inhibit further CaSO_4_ decomposition at elevated temperatures.

**Figure 8:**
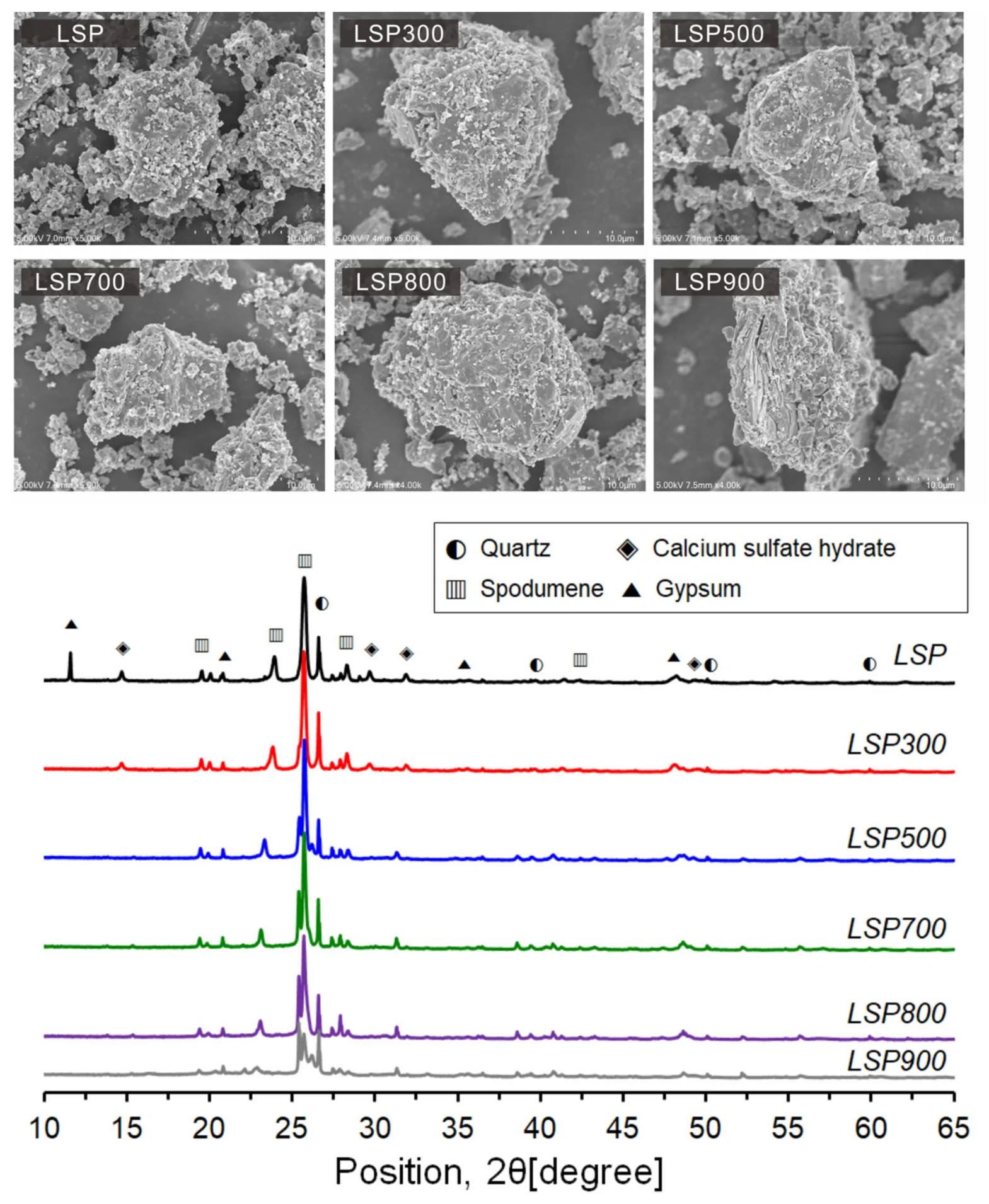
XRD patterns and SEM images of LSP calcined at different temperatures.

Figure 8 shows the XRD patterns and SEM images of LSP at different calcination temperatures. The principal mineral phases identified in LSP are quartz, calcium sulfate hydrates, spodumene, and gypsum. As shown in Figure 8, increasing the calcination temperature leads to a progressive reduction in the diffraction peak intensities of spodumene and sulfate hydrates, whereas quartz—which is thermally stable—retains its main diffraction peaks across the investigated temperature range. Overall, the crystallinity of LSP decreases with increasing calcination temperature. This behavior reflects the temperature- and water-vapor-partial-pressure (pH_2_O)-dependent dehydration-driven phase transitions of gypsum (CaSO_4_·2H_2_O) and bassanite (CaSO_4_·0.5H_2_O) into anhydrite (CaSO_4_). Under low pH_2_O conditions, bassanite can be stabilized at relatively low temperatures, and further heating promotes the transformation into anhydrite. Structurally, the crystal water in gypsum functions as structural water that supports a layered and channel-like order. The characteristic channel-type hydration structure of bassanite is initially produced by partial dehydration, and continued dehydration empties these channels, resulting in contraction and rearrangement into γ-anhydrite. During this process, low-order diffraction reflections associated with layered/channel regularity gradually weaken, whereas characteristic reflections of bassanite and anhydrite become more pronounced, indicating a clear transition in diffraction patterns [48].

Spodumene exhibits a gradual decrease in its principal peak intensities with increasing calcination temperature owing to the accumulation of structural defects and partial amorphization [18]. In the lithium recovery processes, spodumene is thermally treated to expand its lattice volume and subsequently weakened by sulfuric acid, thereby facilitating ion exchange between Li and other cations. The defects accumulated during this process reduce thermal stability, allowing structural collapse to occur even at relatively low temperatures [18,49]. At a calcination temperature of 900 °C, the overall intensities of the crystalline peaks is the lowest among all conditions investigated. This is attributed to the breakdown of aluminosilicate frameworks that leads to the formation of amorphous SiO_2_ and Al_2_O_3_. In the temperature range of approximately 850–900 °C, these amorphous phases undergo partial rearrangement, initiating the early crystallization of mullite. This explains the observed diffraction features [50].

The SEM images on the right side of Figure 8 further illustrate the temperature-dependent morphological evolution of LSP. Uncalcined LSP exhibits a relatively smooth and planar crystalline surface. At 300 °C, partial exfoliation can be observed at the particle edges. Between 500 and 700 °C, surface roughness increases markedly, with accompanied widespread formation of thin flocculent coatings and fine lamellar features [18,51]. Such irregular and defect-rich microstructures are generally associated with enhanced reactivity, suggesting that LSP calcined in the range of 500–700 °C will likely exhibit superior reactivity [52]. At 800 °C, the flocculent structures are partially diminished and rounding of particle edges—indicative of incipient sintering—can be observed. At 900 °C, the particle surfaces are smoother and denser owing to the reformation of plate-like and layered facets, reflecting recrystallization and densification processes [51,53].

**Figure 9:**
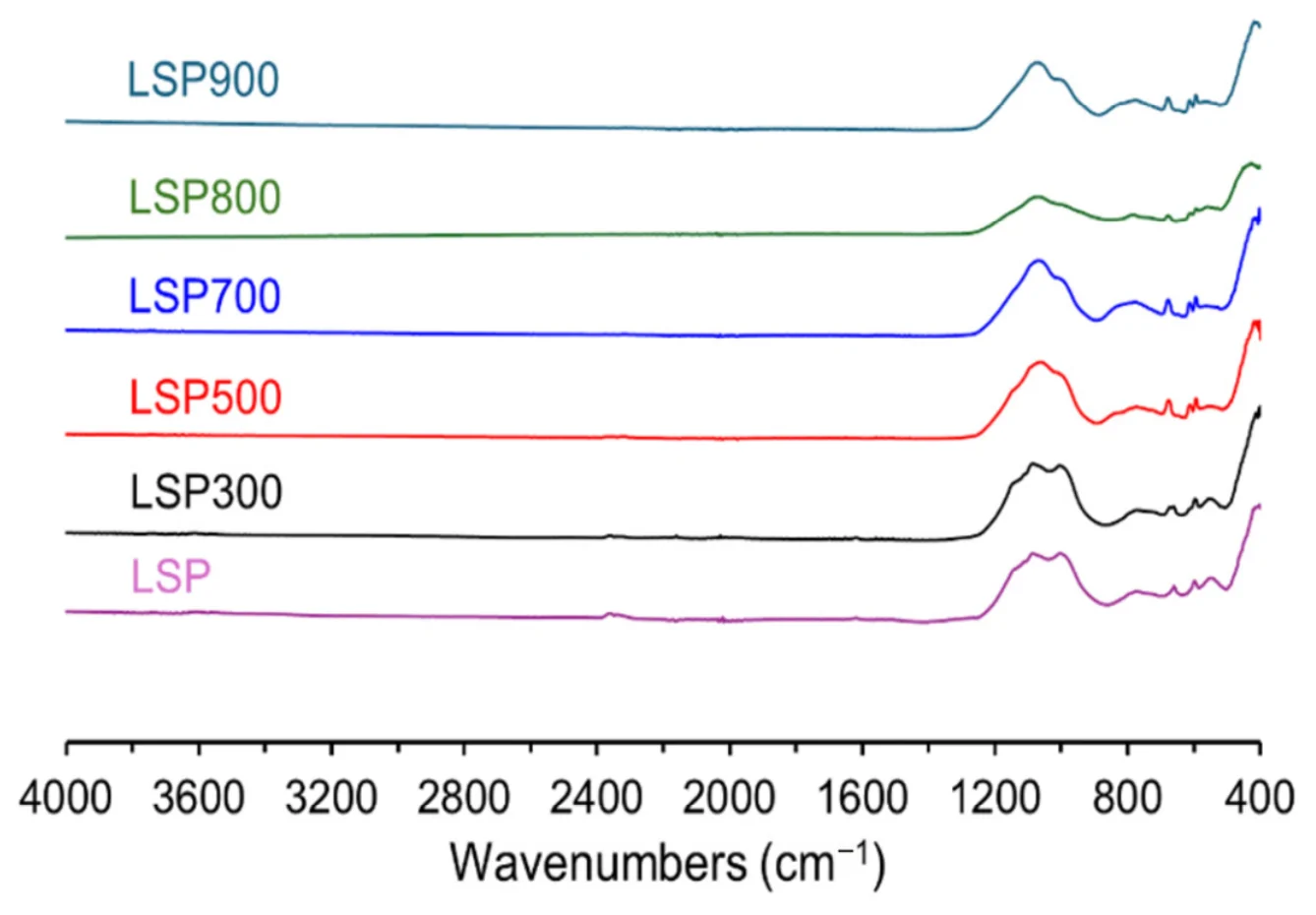
FTIR spectra of LSP calcined at different temperatures.

Figure 9 shows the Fourier-transform infrared (FTIR) spectra of LSP calcined at different temperatures, measured using an FTIR spectrometer (IRXross, Shimadzu Corporation, Kyoto, Japan). Overall, all samples exhibit characteristic absorption bands near 600 cm^−1^ and in the range of 1000–1200 cm^−1^, indicating that LSP is predominantly composed of silicate-based minerals [54]. The band observed around 600 cm^−1^ is attributed to the Si–O–Si bending vibrations and O–H bonding vibrations associated with calcium sulfate hemihydrate [55]. With increasing calcination temperature, a noticeable shift toward lower wavenumbers can be observed in this region, suggesting the decomposition of sulfate-related bonds and progressive loosening of the silicate framework. This behavior is commonly interpreted as a sign of increased amorphous aluminosilicate content at elevated temperatures [18]. The absorption band in the 1000–1200 cm^−1^ region corresponds to the asymmetric stretching vibration of Si–O–T (T = Si or Al) bonds and is widely used as a key indicator for evaluating structural changes in amorphous aluminosilicate networks [24,56]. The vibration band in the range of 1000–1100 cm^−1^ is particularly sensitive to thermal treatment. The main absorption peak of uncalcined LSP is located at 1004 cm^−1^, which shifts as the calcination temperature increases: 1006 cm^−1^ for LSP300, 1058 cm^−1^ for LSP500, and 1066 cm^−1^ for LSP700. This progressive shift and intensification of the band is indicative of enhanced condensation reactions within the Si–O–Al–O framework, which is directly associated with increased alkali reactivity [57]. In contrast, for LSP900, the peak position decreases slightly to 1066 cm^−1^, suggesting the onset of structural rearrangement of amorphous aluminosilicates at high temperatures. This behavior is interpreted as early-stage recrystallization, during which crystalline phases such as mullite or quartz begin to form [54,58].

### 3.2. Hydration Characteristics of LSP-Containing Cement Pastes

**Figure 10:**
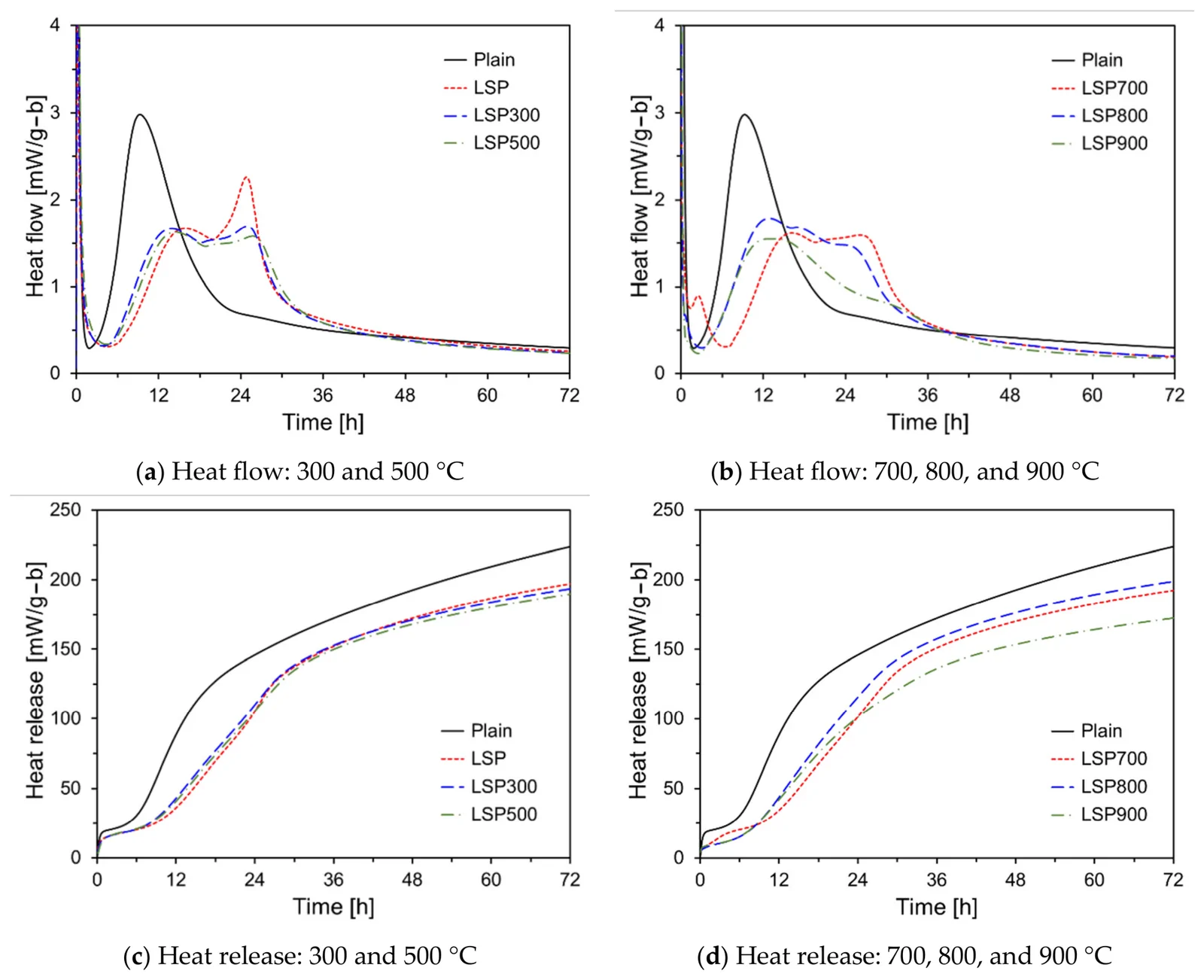
Hydration heat flow and cumulative heat release of samples containing LSP.

In this study, LSP calcined at different temperatures was incorporated at a 30 vol.% replacement level of cement to evaluate the effect of calcination temperature on cement hydration. Figure 10 shows the specific heat flow and cumulative heat release of cement pastes containing LSP calcined at various temperatures. As shown in Figure 10a,b, LSP calcined at 700 °C exhibits the most pronounced retardation. All cement pastes exhibit a rapid heat evolution within approximately 30 min of mixing—which is associated with wetting and initial dissolution reactions—followed by an induction period characterized by relatively low reactivity [59]. During the induction period, a particle network develops through the action of the pore solution, accompanied by the early formation of ettringite (AFt) [60].

The incorporation of LSP prolongs the induction period, thereby delaying early cement hydration. This behavior is primarily attributed to the large specific surface area of LSP [13]. As hydration proceeds, Ca^2+^ ions are preferentially adsorbed onto the LSP surfaces, thereby reducing the ionic concentration in the pore solution. This adsorption suppresses the initial nucleation of Ca(OH)_2_, resulting in delayed hydration and an extended setting time [13]. During the acceleration stage, the intrinsic reactivity of LSP is lower than that of pure cement. Consequently, the number of active hydration sites in the blended system is reduced, leading to a decreased heat release rate [13]. Subsequently, as the hydration of C_3_S and C_2_S becomes dominant, heat evolution increases and the main hydration peak appears after approximately 5–6 h. A secondary peak associated with sulfate depletion—corresponding to the conversion of AFt to AFm, driven by the reactions of C_3_A and C_4_AF—can also be observed [61]. Compared with plain cement paste, the second peak developed later with less pronounced intensity. As shown in Figure 10a,b, the incorporation of LSP prolonged the induction period compared with that of the plain cement paste. However, the induction period generally became shorter as the calcination temperature of LSP increased. In addition, the slope of the heat-flow curve during the acceleration stage increased slightly with increasing calcination temperature, indicating a somewhat faster transition from the induction period to the main hydration reaction.

In addition, a distinct shoulder peak can be observed for the LSP-containing pastes. With increasing calcination temperature, the appearance of this shoulder peak is observed to be progressively delayed with decreasing magnitude. Owing to the relatively high bassanite content of LSP, the shoulder peak is considered to be associated with AFt formation reactions [62]. Compared with plain cement paste, all LSP-containing pastes exhibit lower cumulative heat release, indicating the partial inhibition or dilution of clinker hydration. As the temperature increased, the cumulative heat release up to 72 h for calcined LSP was 197.2, 194.0, 190.0, 192.0, 199.0, and 173.0 J/g, with the highest value observed for LSP calcined at 800 °C. These results suggest that LSP partially suppresses hydration heat evolution or reduces effective clinker reactivity through a dilution effect. In particular, thermally treated LSP at elevated temperatures may exhibit increased crystallinity or reduced chemical reactivity, thus lowering their contribution to hydration reactions. Overall, these findings indicate that the influence of LSP on cement hydration is highly sensitive to both particle characteristics and calcination conditions and that thermal properties such as cumulative heat release respond accordingly.

### 3.3. Potential Reactivity of LSP

#### 3.3.1. R3 Test

In this study, the pozzolanic reactivity of LSP calcined at different temperatures was evaluated using the R3 test in accordance with ASTM C1897. The mixing solution used in the R3 test provides a highly alkaline environment, enabling continuous pozzolanic reactions between the raw material and Ca(OH)_2_. The heat released during this process serves as a direct indicator of pozzolanic reactivity. In general, the hydration heat evolution during the R3 test can be divided into four distinct stages that are analogous to those observed in ordinary cement hydration. The first stage is characterized by high initial heat release occurring within approximately 30 min of mixing owing to the rapid wetting and dissolution of dry materials in the alkaline solution, followed by a dormant period with low reactivity [59,63,64]. The second stage corresponds to AFt formation, induced by the dissolution of reactive SO_4_^2−^, Al^3+^, and CO_3_^2−^ ions during the dormant period [65]. The third stage represents the acceleration phase, during which dissolved amorphous silicate species from the test material react with Ca(OH)_2_, promoting the formation of hydration products such as C–S–H, C–A–S–H, or N-(C)-A–S–H gels. The fourth stage is the deceleration phase, in which the heat release rate decreases as hydration products accumulate. During this phase, residual sulfate and carbonate phases react with AFt to form AFm phases, which are often accompanied by an additional heat evolution peak [59,63,64,65].

**Figure 11:**
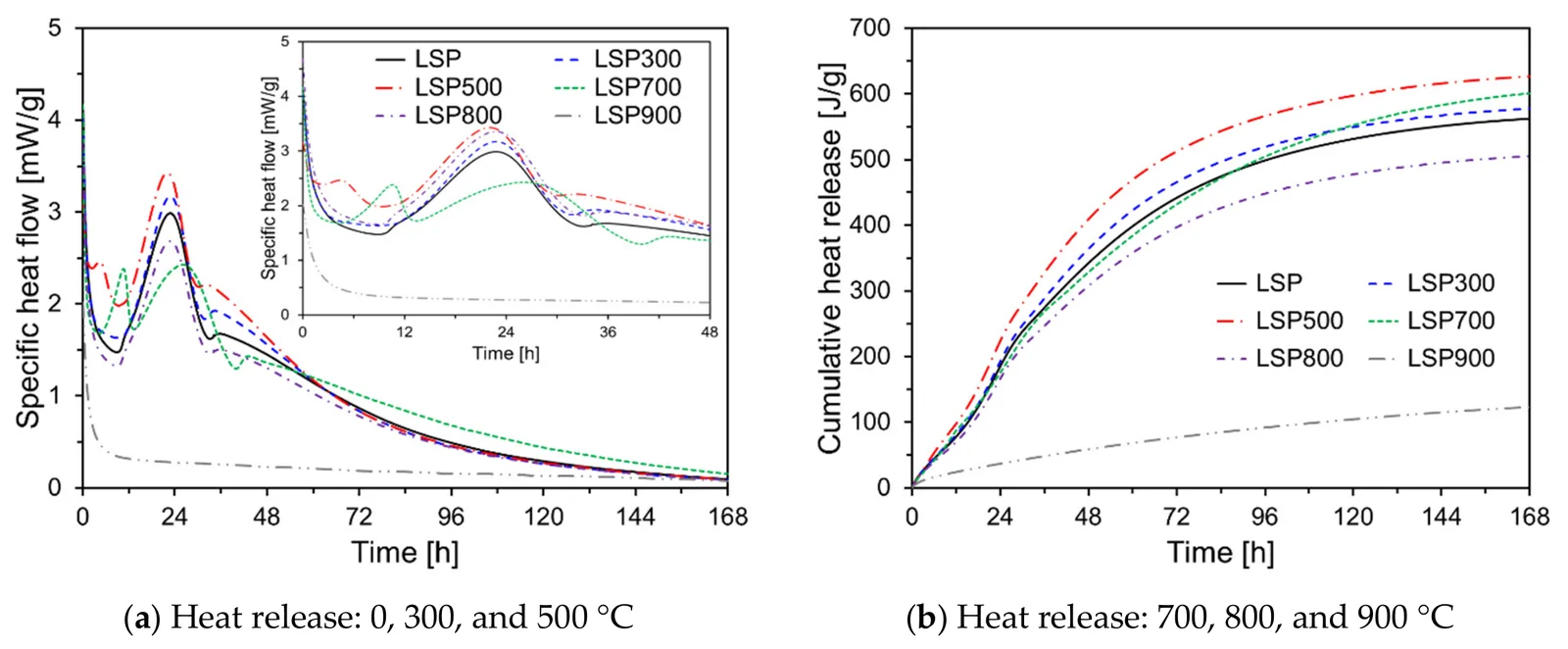
Hydration heat flow and cumulative heat release results of R3 testing.

Figure 11 shows the specific heat flow and cumulative heat release obtained from the R3 testing. As shown in Figure 11a,b, all samples exhibit main hydration reaction peaks between approximately 20–35 h. Among the tested materials, LSP500 exhibits the highest specific heat flow peak of 3.43 mW/g, indicating the highest pozzolanic reactivity. This enhanced reactivity is attributed to the increased amorphous content generated under optimal calcination conditions, which activates hydration reactions [11]. The cumulative heat release values for LSP calcined at 0, 300, 500, 700, 800, and 900 °C are 562.2, 541.7, 626.6, 600.9, 505.2, and 123.1 J/g, respectively, with LSP500 showing the highest cumulative heat release. In contrast, LSP900 exhibits a pronounced reduction in reactivity, indicating that an excessive calcination temperature adversely affects pozzolanic performance. According to the classification proposed by Kalina et al., materials with cumulative heat release below 100 J/g SCM are considered inert, those in the range of 100–200 J/g SCM exhibit moderate pozzolanicity, and materials exceeding 200 J/g SCM are classified as highly pozzolanic [11,66,67]. Based on this criterion, all calcined LSP samples—except LSP900—satisfy the requirements for highly pozzolanic materials. In particular, calcination at 500 °C induces the highest cumulative heat release (626.6 J/g), indicating superior pozzolanic reactivity. This behavior is attributed to the formation of abundant amorphous silica during calcination, which readily reacts with Ca(OH)_2_ to promote the formation of the C–S–H and C–A–S–H phases, thereby accelerating hydration reactions [63]. Consequently, rapid formation of hydration products and increased cumulative heat release reflect the enhanced reactivity of LSP calcined at 500 °C [63,68,69].

**Figure 12:**
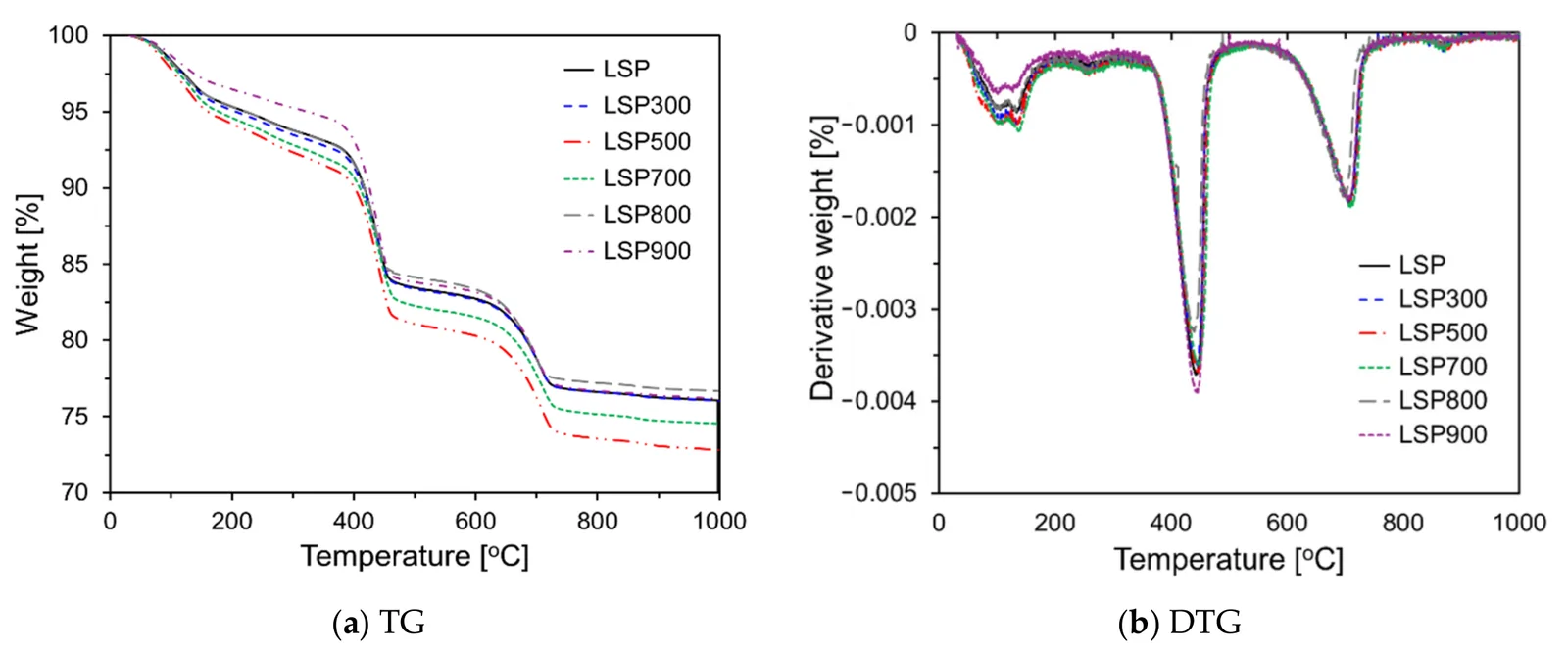
TG–DTG results for LSP calcined at different temperatures.

For LSP calcined at different temperatures, paste specimens were prepared following the same procedure as the R3 test specified in ASTM C1897 and cured at 40 °C for 7 d. TG–DTG analyses were subsequently conducted to characterize the hydration products, and the results are presented in Figure 12. As shown in Figure 12, distinct mass losses can be observed in the temperature ranges of 50–200, 375–475, and 600–800 °C. The mass loss in the range of 50–200 °C (M_50–200_) is primarily attributed to the dehydration of C–S–H gel and AFt, whereas the mass loss between 375 and 475 °C corresponds to the decomposition of Ca(OH)_2_. The mass loss observed between 600 and 800 °C is associated with the decomposition of CaCO_3_ [70,71,72]. Among the tested samples, LSP500 exhibits the largest mass loss in the 50–200 °C range. LSP500 is commonly used as an indirect indicator of gel accumulation [73]. As presented in Table 3, the bound water content reaches a maximum value of 8.38% at a calcination temperature of 500 °C. This indicates the greatest accumulation of hydration products and suggests that the amorphization of Si/Al phases induced by calcination effectively promotes the formation of bound water [74,75]. In addition, a lower mass loss fraction associated with Ca(OH)_2_ decomposition in the range of 375–475 °C indicates greater consumption of Ca(OH)_2_ through pozzolanic reactions [70]. In the case of LSP500, the Ca(OH)_2_ content was 28.89%, which is the lowest among all variables, demonstrating that Ca(OH)_2_ consumption owing to pozzolanic reactions was most pronounced at 500 °C. These findings are consistent with those of previous studies reporting that thermally treated LSP enhances secondary reactions with Ca(OH)_2_, thereby promoting pozzolanic activity [74].

In this study, the bound water content of the specimens used for the TG–DTG analysis was quantified using Equation (1); the results are presented in Table 4.

(1) $$\mathrm{Bound}\;\mathrm{water}\;=\frac{W_{0}-W_{350\;{}^{\circ}C}\;}{W_{0}-W_{c}}\times 100,$$

where $W_{c}$ denotes the mass of the crucible prior to heating; $W_{350^{\circ }C}$ represents the combined mass of the crucible and sample after heating to 350 °C; and $W_{0}$ corresponds to the mass of the sample, including the empty crucible before heating.

**Figure 13:**
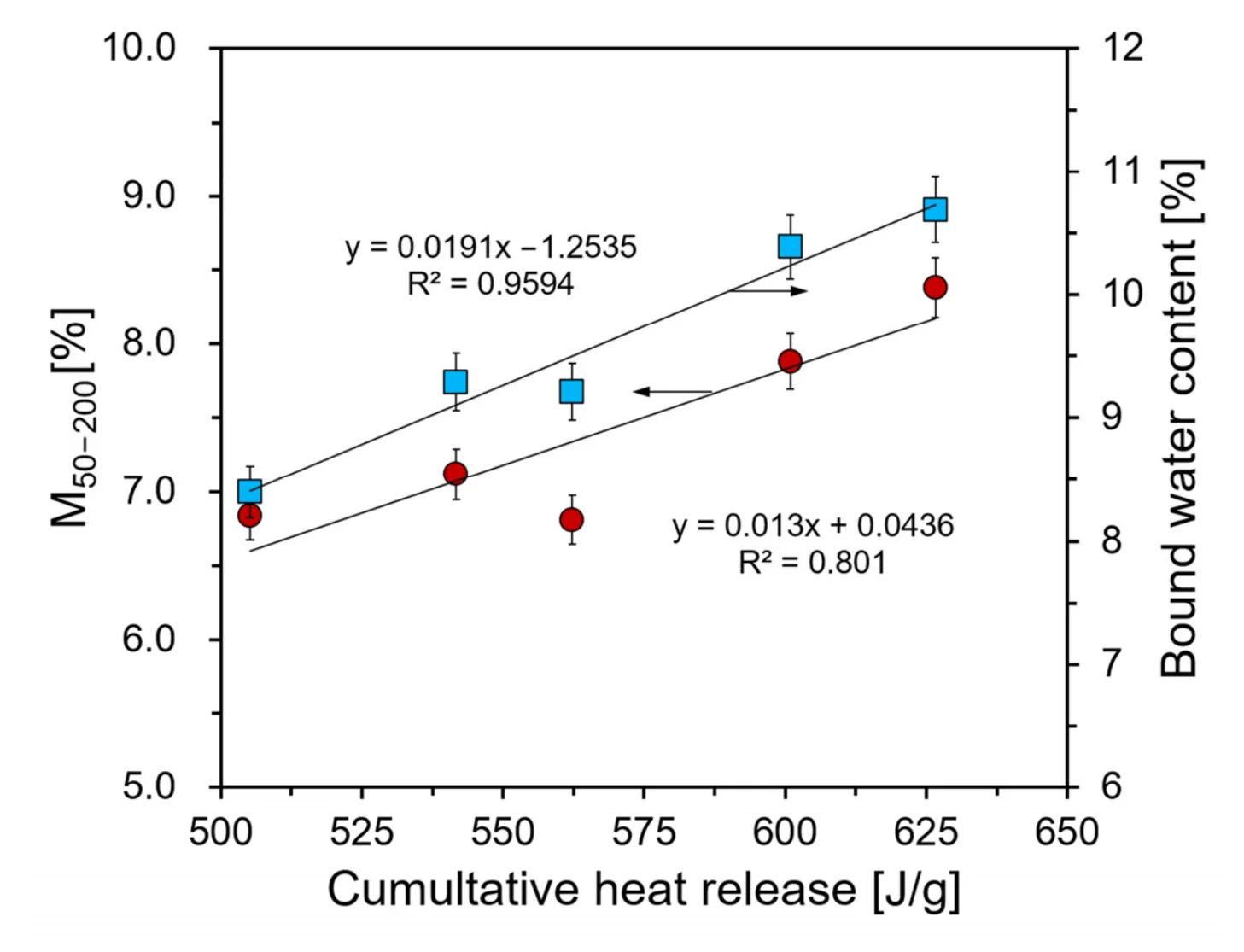
Correlation between cumulative heat release and both M_50–200_ and bound water content at 7 d, obtained via R3 testing.

The bound water content obtained from the R3-based paste specimens reached a maximum value of 10.69% for LSP500 and a minimum value of 6.83% for LSP900. These values quantitatively reflect the accumulation of major hydration products formed during hydration, namely the C–S–H, AFt, and AFm phases [65]. To verify the consistency between the TG–DTG-derived indicators and calorimetric results, Figure 13 shows the correlations between cumulative heat release and both M50–200 and bound water content. Linear regression analysis yielded a coefficient of determination of $R^{2}=0.801$ for the relationship between cumulative heat release and M50–200, and a higher coefficient of $R^{2}=0.9594$ for the relationship between cumulative heat release and bound water content, indicating strong correlations between these parameters. These results demonstrate that the R3 test effectively isolates the intrinsic reactivity of SCMs by simulating a cement hydration environment in which particle packing effects are minimized, thereby enabling a clear assessment of SCM-specific reactions [76]. Furthermore, the superior correlation and higher bound water content observed at the calcination temperature of 500 °C indicate that initial gel formation and pozzolanic reactions were more active under this thermal activation condition than at other calcination temperatures.

#### 3.3.2. Chapelle Test

**Figure 14:**
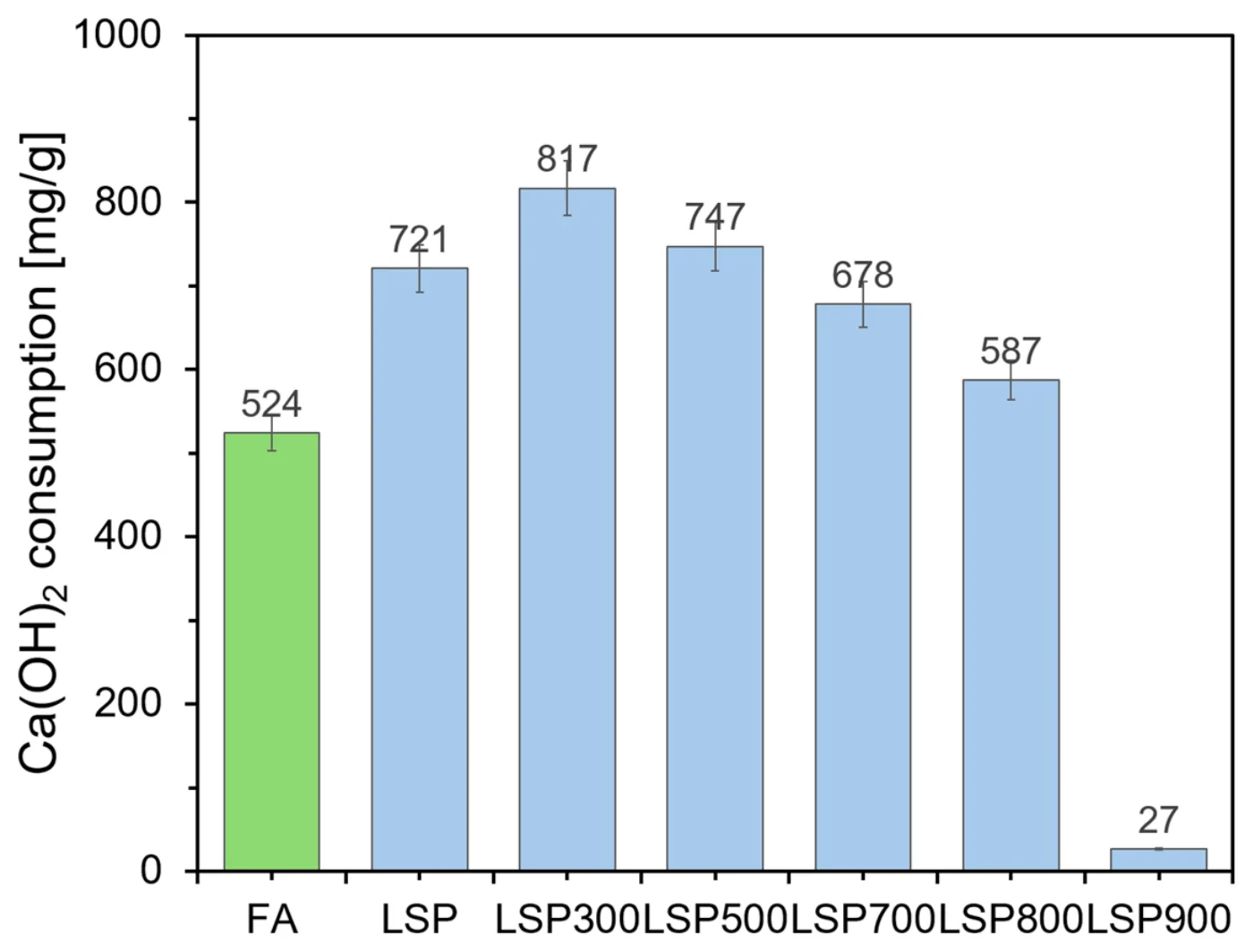
Chapelle test results for FA and LSP as a function of calcination.

Figure 14 shows the results of the modified Chapelle test for LSP and FA as a function of calcination temperature. This test quantitatively evaluates the amount of Ca(OH)_2_ consumed by a material under aqueous conditions. As shown in Figure 14, the Ca(OH)_2_ consumption of untreated LSP reaches 721 mg/g, which is approximately 1.38 times higher than that of FA (524 mg/g)—a widely used conventional pozzolanic material. With respect to temperature-dependent reactivity, LSP300 exhibits a Ca(OH)_2_ consumption of 817 mg/g, representing a 1.13-fold increase compared to that of untreated LSP. That of LSP500 is 747 mg/g, which is comparable to, but slightly higher than, that of untreated LSP. In contrast, when the calcination temperature exceeds 700 °C, the Ca(OH)_2_ consumption decreases to values lower than that of untreated LSP, and LSP900 exhibits behavior corresponding to an essentially inert material. This trend is consistent with the stepwise dehydration and phase transformation of gypsum during heating—progressing from gypsum to bassanite, subsequently to soluble anhydrite III, and finally to insoluble anhydrite II [77,78,79]. In LSP, anhydrite III is predominantly formed in the calcination temperature range of approximately 300–500 °C and gradually transforms into insoluble anhydrite II as the temperature increases. This temperature range is in good agreement with the formation and transformation window of anhydrite III (approximately 94–400 °C) reported by Krejsová et al. [78,79].

The increased Ca(OH)_2_ consumption observed in the 300–500 °C range is attributed to structural rearrangement and densification occurring during the transition from anhydrite III to anhydrite II [79]. This transformation is accompanied by internal lattice contraction and microcrack formation, which transiently expose new reactive surfaces and enhance the reactivity toward Ca(OH)_2_ in aqueous environments [80,81]. Conversely, at calcination temperatures above 700 °C, progressive sintering of anhydrite II occurs, leading to the loss of planar crystal surfaces, pore closure, and particle coarsening. These changes reduce the effective specific surface area and number of active reaction sites, thereby decreasing Ca(OH)_2_ consumption [78,79]. Accordingly, enhanced reactivity dominated by microcrack formation can be observed in the 300–500 °C range, whereas sintering-induced deactivation becomes predominant at temperatures of 700 °C and above.

#### 3.3.3. Leaching Test

**Figure 15:**
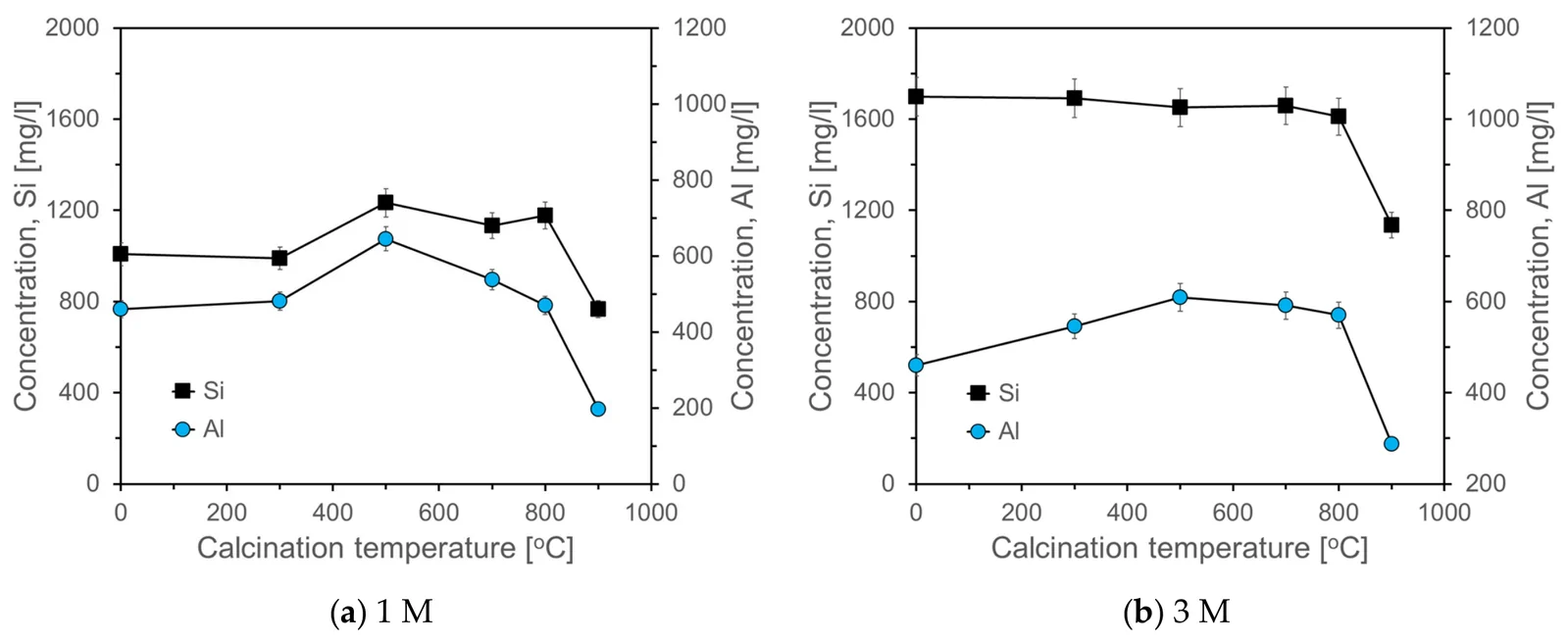

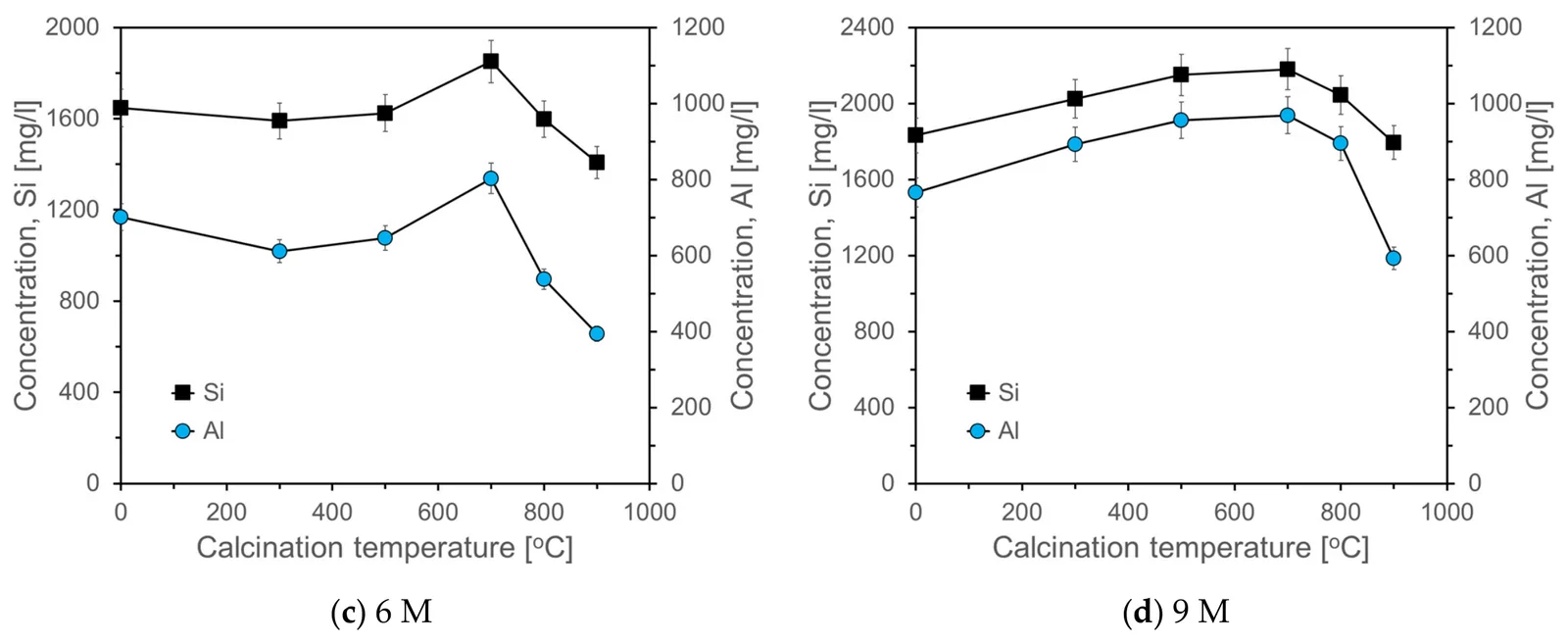
Dissolution behavior of Si and Al for LSP calcined at different temperatures in various NaOH solution concentrations.

Figure 15 shows the variations in Si and Al dissolution concentrations of LSP as a function of calcination temperature in NaOH solutions with concentrations of 1, 3, 6, and 9 M. With increasing NaOH concentration, the concentrations of dissolved Si and Al ions consistently increases. This can be attributed to the enhanced cleavage of Si–O and Al–O bonds caused by higher OH^−^ ion availability [82,83]. However, the dissolution trends of Si and Al with respect to calcination temperature vary depending on the NaOH concentration.

For the 1 M NaOH solution, the Si concentration remains relatively constant up to a calcination temperature of 300 °C and increases sharply at 500 °C. This behavior indicates that structural water dehydration and amorphous phase formation occur near 500 °C, leading to the partial disruption of Si–O–Al bonds and enhanced dissolution [84,85]. At higher calcination temperatures, the Si concentration gradually decreases. The dissolution trend of Al follows a pattern similar to that of Si, although the average Al concentration is approximately 580 mg/L lower than that of Si. Overall, increasing the NaOH concentration resulted in an increased dissolution of both Si and Al. For the 3 M NaOH solution, the Si concentration remains consistently higher than that of Al at all calcination temperatures. The Si concentration remains nearly constant at approximately 1660 mg/L up to 800 °C, followed by a sharp decrease to 1135 mg/L at 900 °C. In contrast, the Al concentration continuously increases up to 609 mg/L at 500 °C, after which it decreases, exhibiting a sharp decline at 900 °C. This is similar behavior to that observed for Si. For the 6 M NaOH solution, the maximum concentrations of dissolved Si and Al are observed at a calcination temperature of 700 °C, reaching 1851 and 803 mg/L, respectively. Above 700 °C, the concentration of dissolved ions markedly decreases. For the 9 M NaOH solution, the Si concentration increases steadily with increasing calcination temperature reaching the maximum in the range of 500–700 °C, followed by a subsequent decrease. A similar trend is observable for Al dissolution.

For all NaOH concentrations, the dissolution experiments indicate that the highest Si and Al concentrations occurred in the calcination temperature range of 500–700 °C. This behavior is attributed to structural relaxation and enhanced dissolution resulting from amorphous aluminosilicate phase formation [24]. In contrast, at 900 °C, the concentrations of dissolved Si and Al decreased sharply for all NaOH concentrations. This reduction is attributed to excessive thermal treatment that induces recrystallization and chemically stable crystalline phase formation, thereby stabilizing the Si–O–Al bonds and reducing dissolution reactivity [24]. These results are consistent with the findings reported by Zhao et al. for ferronickel slag, which showed similar dissolution trends as a function of NaOH concentration [83]. Although increasing the NaOH concentration generally accelerates Si and Al dissolution at exceedingly high alkalinities, the dissolved ions may react with Na^+^ to form N–A–S–H or C–A–S–H gels, thereby reducing the concentrations detected in solution. In addition, Wang et al. reported that even when the amorphous content of LSP increases at calcination temperatures above 900 °C, the overall reactivity decreases owing to the formation of stable silicoaluminate crystalline phases [44].

### 3.4. Compressive Strength Results

**Figure 16:**
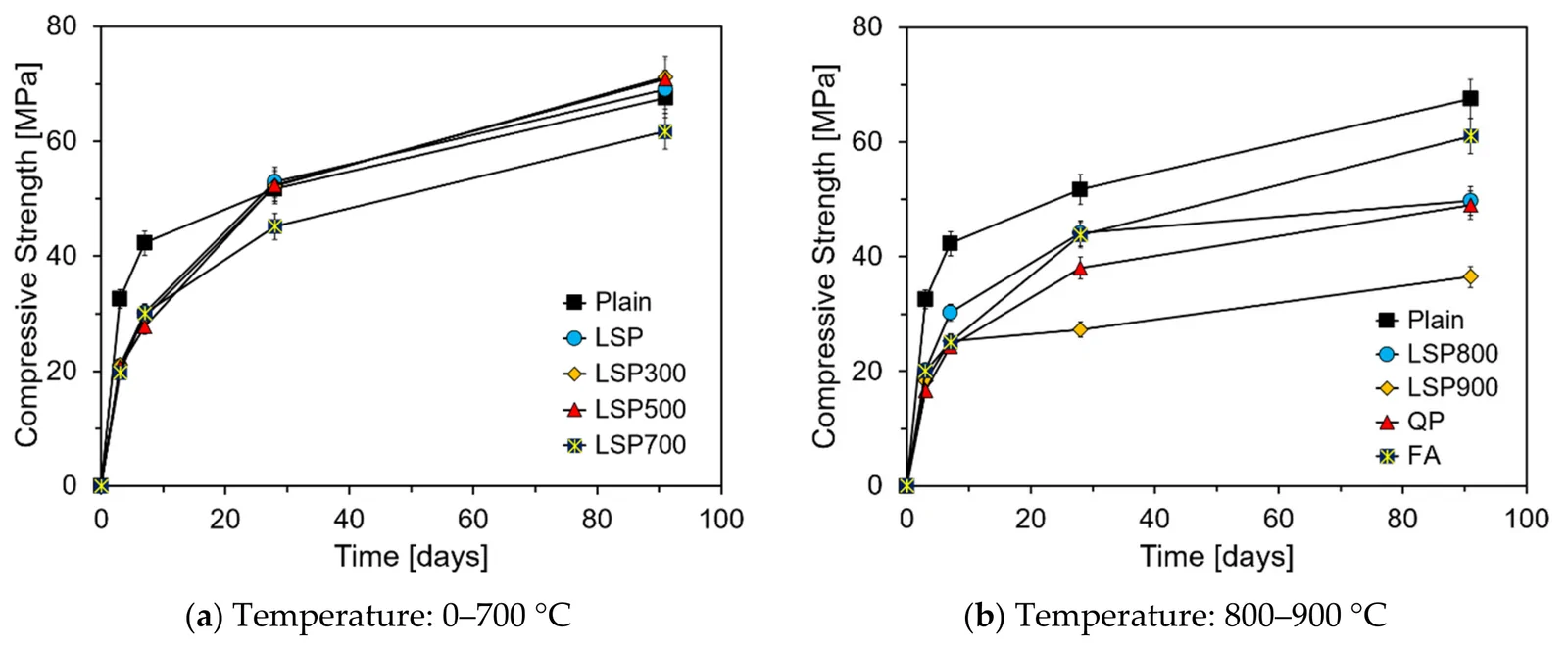
Compressive strengths of the mortar specimens.

Figure 16 shows the compressive strengths of the mortars at different curing ages. For all mixtures, the compressive strength can be observed to increase continuously with curing age. The Plain mortar samples achieve 3 and 7 d compressive strengths of 32.6 and 42.3 MPa, respectively. In contrast, mortars incorporating LSP exhibit lower early-age strengths, with 3 and 7 d values ranging from 18.3 to 21.9 and 25.3–30.3 MPa, respectively. This behavior indicates that at early ages, the intrinsic reactivity of LSP is relatively low and that dilution effects dominate the hydration process [86]. For the mortar containing the inert material (QP), the 3 and 7 d compressive strengths reach 16.7 and 24.4 MPa, respectively, whereas those of FA are 17.3 and 25.1 MPa. As QP is essentially nonreactive, its contribution to strength development is limited to the physical filler effect [86,87]. Compared with FA, the LSP-containing mortars exhibited comparable or superior early age strength enhancement. This is attributable to the filler effect and provision of nucleation sites. In addition, the relatively high sulfate content in LSP is thought to promote AFt formation, further contributing to early-age strength development [43,88].

At 28 d, a different trend can be observed. The compressive strengths of mortars containing uncalcined LSP, LSP calcined at 300 °C, and LSP calcined at 500 °C are 52.9, 52.2, and 52.4 MPa, respectively, exceeding that of the Plain mortar (51.7 MPa). In contrast, mortars incorporating LSP calcined at 700–800 °C exhibited 28 d compressive strengths of approximately 86% of that of the Plain mortar, whereas the strength of the LSP900 mixture decreased markedly to approximately 53% of the Plain value. The 28 d compressive strength of FA-containing mortar is 43.8 MPa, comparable to that of the LSP800 mixture. At 91 d, these trends become more pronounced. Mortars containing uncalcined LSP and LSP calcined at 300 and 500 °C exhibit higher compressive strengths than the Plain mortar. In particular, LSP300 and LSP500 achieve the highest 91 d compressive strengths at 70.9 and 71.1 MPa, respectively, which are approximately 6–10% higher than those of the Plain (67.2 MPa) and uncalcined LSP (65.3 MPa) mortars. These results indicate that thermal activation in the temperature range of 300–500 °C provides the most favorable conditions for enhancing the reactivity [87]. In contrast, mortars incorporating LSP calcined at temperatures of 800 °C and above exhibit a pronounced reduction in strength development beyond 28 d. LSP900 shows the lowest compressive strengths, with values of 27.3 MPa at 28 d and 36.6 MPa at 91 d. This strength deterioration is attributed to excessive calcination, which induces the recrystallization of amorphous phases and the formation of inert crystalline phases, thereby significantly reducing LSP reactivity [87].

**Figure 17:**
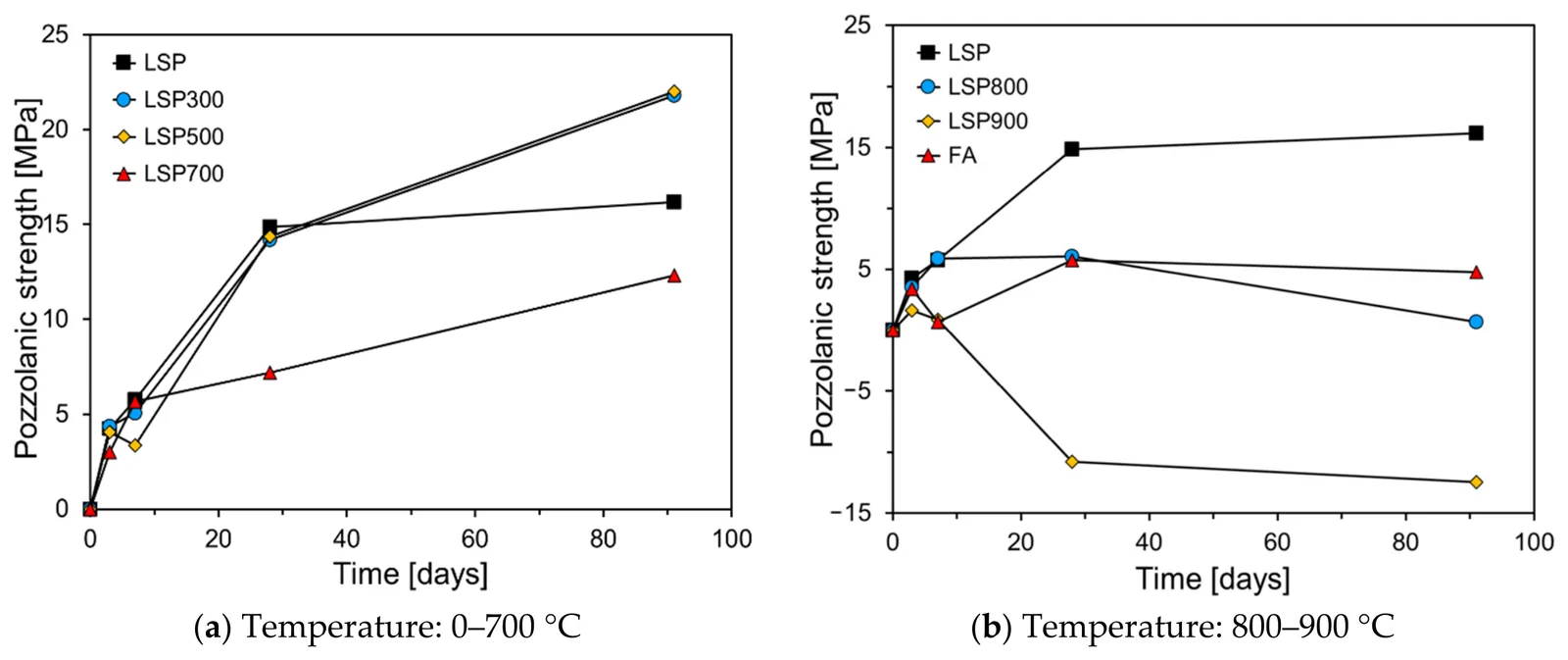
Evolution of the pozzolanic strengths of mortars at different curing ages.

Figure 17 shows the evolution of the pozzolanic strength of the mortars at different curing ages. The pozzolanic strength was calculated as the difference in compressive strengths of the SCM-containing mortar and reference mortar incorporating an inert material (QP) but having an identical particle size distribution. As shown in Figure 17, the slope of compressive strength enhancement at 3 d indicates that the observed strength differences at this age are primarily attributable to the physical filler effects associated with nucleation site provision rather than true pozzolanic reactions. Accordingly, to isolate the chemical contribution of pozzolanic activity, this study defined pozzolanic strength as the difference between the post-3 d strength increment of each mixture and that of the inert QP reference.

At 7 d, the pozzolanic strengths of mortars incorporating LSP and calcined at 700 and 800 °C are observed to be relatively high, reaching 2.68 and 2.37 MPa, respectively. In contrast, the remaining LSP-containing mortars—except for the 900 °C condition—exhibit a low average pozzolanic strength of 0.57 MPa, whereas FA has a negligible value of 0.07 MPa. The LSP900 mortar has a lower compressive strength than that of the QP reference, indicating a detrimental effect on cement hydration and nearly negligible pozzolanic activity. This behavior is attributed to excessive thermal activation that induces the recrystallization of amorphous phases and a consequent reduction in pozzolanic reactivity [47]. Based on the 7 d results, higher calcination temperatures appear to accelerate the onset of pozzolanic reactions, whereas mortars with lower calcination temperatures and FA initiate pozzolanic activity at around 7 d. This observation is consistent with that of previous studies reporting that the pozzolanic reaction of FA typically begins between 3 and 7 d [34]. At 28 d, FA can be observed to exhibit a pozzolanic strength of 5.1 MPa, whereas mortars containing uncalcined LSP and LSP calcined at 300 and 500 °C have substantially higher values of 9.7, 9.8, and 10.3 MPa, respectively. In contrast, the LSP700 mortar—which exhibits the highest pozzolanic strength at 7 d—has a reduced value of 4.2 MPa at 28 d, corresponding to approximately 41% that observed for LSP500. LSP800 has a pozzolanic strength similar to that observed at 7 d. At 91 d, the highest pozzolanic strength can be observed for LSP500 (17.9 MPa), followed closely by LSP300 (17.5 MPa). Although the 28 d pozzolanic strengths of the LSP300–500 mixtures are comparable to that of the uncalcined LSP, their 91 d strengths exceed that of the uncalcined LSP (14.2 MPa), indicating that thermal activation enhances long-term pozzolanic activity. The pozzolanic strength of FA at 91 d is 11.6 MPa, which is lower than that of all LSP mixtures calcined between 300 and 500 °C.

These results suggest that the sulfate- and silica-rich composition of LSP contributes to long-term strength development through reactions with Ca(OH)_2_ during cement hydration, promoting the formation of AFt and C–S–H gel [88]. The presence of Ca-bearing phases further stabilizes AFt, increases hydration product volumes, and reduces porosity, thereby enhancing strength. This behavior is consistent with the trends reported for limestone-containing cementitious systems [43,88]. In contrast, the pozzolanic strength of the LSP700 mixture at 91 d at 9.3 MPa is lower than that of FA, whereas the LSP800 and LSP900 mortars display negative values, indicating a reduction in compressive strength and suppressed pozzolanic activity. Overall, the results demonstrate that the pozzolanic reactivity of the LSP used in this study exceeds that of FA and can be effectively enhanced through thermal activation in the temperature range of approximately 300–500 °C. Conversely, excessive calcination at high temperatures (approximately 700–900 °C) leads to reduced pozzolanic reactivity and adversely effects cement hydration [47]. These pozzolanic strength trends are consistent with the results obtained from the Chapelle test and alkaline leaching experiments.

## 4. Conclusions

This study systematically evaluated the temperature-dependent evolution of LSP calcined over a wide temperature range of 300–900 °C and quantitatively assessed the influence of these structural transformations on cement hydration reactivity. To clarify the applicability of thermally treated LSP as an SCM, its pozzolanic reactivity was comprehensively investigated as a function of calcination temperature. The main findings are summarized as follows.

LSP is a complex mineral assemblage composed of quartz, spodumene, sulfate-bearing phases, and carbonate-derived minerals, with a chemical composition dominated by SiO_2_ and Al_2_O_3_. This composition is comparable to that of conventional pozzolanic materials such as FA. Partial amorphization occurring during lithium extraction imparts intrinsic pozzolanic reactivity to LSP; however, its relatively low CaO content limits its use as a standalone binder. During thermal treatment, sulfate hydrates progressively dehydrate into bassanite and anhydrite, while spodumene gradually loses crystallinity due to defect accumulation.

R3 calorimetry results indicated a typical four-stage hydration pattern, with the main reaction peak occurring at approximately 20–35 h. The cumulative heat release varied significantly with calcination temperature, reaching a maximum for LSP calcined at 500 °C. This behavior indicates that, at this temperature, pozzolanic reactions with Ca(OH)_2_ were most effectively promoted by the amorphous silicate phases generated during calcination, resulting in enhanced C–S–H and C–A–S–H gel formation. Consistent trends were also observed via TGA, confirming the reliability of the calorimetric assessment.

Modified Chapelle test results showed that LSP calcined at 300 and 500 °C had Ca(OH)_2_ consumptions of 817 and 747 mg/g, respectively, exceeding that of untreated LSP. In contrast, Ca(OH)_2_ consumption gradually decreased at calcination temperatures above 700 °C, reaching an essentially inert level at 900 °C. In the 300–500 °C range, enhanced reactivity was attributed to microcrack formation and structural rearrangement, whereas sintering dominated at temperatures ≥700 °C, leading to a marked reduction in pozzolanic activity.

Alkaline leaching experiments revealed that Si and Al dissolution generally increased with NaOH concentration, with maximum dissolution observed for LSP calcined at 500–700 °C. This behavior reflects the pronounced structural relaxation associated with amorphous phase formation. In contrast, LSP calcined at 900 °C exhibited a sharp decrease in Si and Al dissolution due to recrystallization and the formation of thermodynamically stable crystalline phases, which stabilized the Si–O–Al framework and suppressed reactivity.

Mortars incorporating LSP exhibited lower early-age strengths than plain OPC mortar due to dilution effects and limited early reactivity. However, compared with FA, LSP-containing mortars showed comparable or superior performance owing to filler-induced nucleation effects and sulfate-assisted AFt formation. At 28 and 91 d, mortars with LSP calcined at 300 and 500 °C demonstrated higher compressive and pozzolanic strengths than both plain OPC and FA-containing mortars, confirming that this temperature range provides optimal thermal activation. In contrast, LSP calcined at 700–900 °C exhibited significantly reduced long-term strength and pozzolanic activity due to recrystallization and formation of inert phases, indicating that excessive high-temperature treatment adversely affects LSP reactivity.

Overall, the results demonstrate that LSP possesses higher pozzolanic potential than FA and that its reactivity can be substantially enhanced through controlled thermal activation at approximately 300–500 °C. Conversely, excessive calcination at higher temperatures (≥700 °C) leads to reactivity loss and unfavorable hydration behavior. These conclusions are consistent with trends observed in Chapelle testing, alkaline leaching experiments, calorimetry, and mechanical performance, providing a coherent framework for optimizing the thermal activation of LSP for use as an SCM.

The main limitations of this study are the use of LSP obtained from a single production source, fixed laboratory-scale calcination conditions, and a single cement replacement level. In addition, the performance evaluation mainly focused on hydration characteristics and compressive strength. Further research should therefore examine the variability of LSP from different sources, optimize the replacement ratio and calcination parameters, and evaluate long-term durability, volume stability, environmental safety, and the life-cycle impacts of thermal activation.

## Figures and Tables

**Table 1:** Chemical compositions and densities of the raw materials.

	Chemical Composition (wt.%)											Density (g/cm^3^)
	SiO_2_	Al_2_O_3_	SO_3_	CaO	Fe_2_O_3_	K_2_O	MgO	P_2_O_5_	Na_2_O	MnO	TiO_2_	
OPC	21.6	5.2	2.2	61.3	3.1	1.1	2.3	-	0.1	-	-	3.14
LSP	54.42	23.11	11.32	6.30	2.29	0.96	0.55	0.38	0.34	0.34	-	2.50
FA	54.48	19.80	1.63	7.70	8.05	1.13	2.25	0.83	2.75	-	1.03	2.42

**Table 2:** Chemical composition of LSP calcined at different temperatures.

	Chemical Composition (wt.%)											
	SiO_2_	Al_2_O_3_	SO_3_	CaO	Fe_2_O_3_	K_2_O	MgO	P_2_O_5_	MnO	TiO_2_	ZnO	Cr_2_O_3_
LSP	54.42	23.11	11.32	6.30	2.29	0.96	0.55	0.38	0.34	-	2.50	0.03
LSP300	54.45	23.16	10.92	6.33	2.57	0.98	0.47	0.39	0.43	0.08	0.04	0.02
LSP500	54.67	23.28	10.75	6.24	2.56	0.97	0.47	0.41	0.37	0.05	0.04	0.02
LSP700	55.16	23.52	9.88	6.19	2.62	1.04	0.47	0.41	0.43	0.06	0.04	0.03
LSP800	55.85	23.86	8.82	6.26	2.59	1.05	0.48	0.39	0.39	0.07	0.04	0.02
LSP900	57.10	24.44	6.62	6.38	2.65	1.12	0.53	0.42	0.43	0.08	0.04	0.03

**Table 3:** M_50–200_ and Ca(OH)_2_ contents of the cement pastes.

	LSP	LSP300	LSP500	LSP700	LSP800	LSP900
M_50–200_ [%]	6.81	7.12	8.38	7.88	6.84	5.24
Ca(OH)_2_ [%]	29.54	29.25	28.89	29.47	29.11	28.28

**Table 4:** Cumulative heat release and bound water results obtained from the R3 test.

	Cumulative Heat Release (J/g of SCM)	Bound Water (g/100 g of Dried Paste)
LSP	562.2	9.21
LSP300	541.7	9.29
LSP500	626.6	10.69
LSP700	600.9	10.39
LSP800	505.2	8.40
LSP900	123.1	6.83

## Data Availability

The original contributions presented in this study are included in the article. Further inquiries can be directed to the corresponding author.
